# Inhibition of Tumor Microenvironment-Driven JAK-STAT Signaling Enhances Response to Arginine Deprivation Therapy in Triple-Negative Breast Cancer

**DOI:** 10.3390/cells15010025

**Published:** 2025-12-23

**Authors:** Hila Tishler, Shahar Ziman, Kuoyuan Cheng, Kun Wang, Neel Sanghvi, Lital Adler, Gil Stelzer, Hillary Maniriho, Bareket Dassa, Elizabeta Bab-Dinitz, Michal Levi, Sivan Galai, Omer Goldman, Yarden Ariav, Naama Darzi, Saar Ezagouri, Nitsan Nimni, Nataly Rosenfeld, Ron Rotkopf, Alexander Brandis, Tevie Mehlman, Roni Oren, Mirie Zerbib, Yuri Kuznetsov, Sara Donzelli, Giovanni Blandino, Rony Seger, Eytan Ruppin, Ayelet Erez

**Affiliations:** 1 Department of Molecular Cell Biology, Weizmann Institute of Science, P.O. Box 26, Rehovot 76100, Israel; hila.tishler@weizmann.ac.il (H.T.); shahar.ziman@weizmann.ac.il (S.Z.); lital.adler@weizmann.ac.il (L.A.); elizabeta.bab-dinitz@weizmann.ac.il (E.B.-D.); sivangalai@gmail.com (S.G.); omer.goldman@metabocure.com (O.G.); yarden.ariav@weizmann.ac.il (Y.A.); naama.darzi@weizmann.ac.il (N.D.); sezagouri@gmail.com (S.E.); nitsan.nimni@weizmann.ac.il (N.N.); nataly.rosenfeld@weizmann.ac.il (N.R.); 2 Data Science Laboratory, Center for Cancer Research, National Cancer Institute, National Institutes of Health, Bethesda, MD 20892, USA; cheng.ky@hotmail.com (K.C.); neel.sanghvi@nih.gov (N.S.); eyruppin@gmail.com (E.R.); 3 Departments of Comparative Biosciences and Bioengineering, The Cancer Center at Illinois, University of Illinois Urbana-Champaign, Urbana, IL 61802, USA; kwang222@illinois.edu; 4 Department of Life Sciences Core Facilities, Weizmann Institute of Science, P.O. Box 26, Rehovot 76100, Israel; gil.stelzer@weizmann.ac.il (G.S.); bareket.dassa@weizmann.ac.il (B.D.); ron.rotkopf@weizmann.ac.il (R.R.); alexander.brandis@weizmann.ac.il (A.B.); tevie.mehlman@weizmann.ac.il (T.M.); 5 Felsenstein Medical Research Center, Gray Faculty of Medical and Health Sciences, Tel Aviv University, Petah Tikva 49100, Israel; hillarym@mail.tau.ac.il; 6 Department of Veterinary Resources, Weizmann Institute of Science, P.O. Box 26, Rehovot 76100, Israel; roni.oren@weizmann.ac.il (R.O.); mirie.zerbib@weizmann.ac.il (M.Z.); yuri.kuznetsov@weizmann.ac.il (Y.K.); 7 Translational Oncology Research Unit, Istituto di Ricovero e Cura a Carattere Scientifico (IRCCS), Regina Elena National Cancer Institute, 00144 Rome, Italy; sara.donzelli@ifo.it (S.D.); giovanni.blandino@ifo.it (G.B.); 8 Department of Immunology and Regenerative Biology, Weizmann Institute of Science, P.O. Box 26, Rehovot 76100, Israel; rony.seger@weizmann.ac.il

**Keywords:** ASS1, arginine, tumor microenvironment, JAK-STAT

## Abstract

**Highlights:**

**What are the main findings?**

- Arginine depletion suppresses TNBC cell growth in vitro but not in vivo, due to a TME-mediated arginine supply and JAK-STAT activation.
- ASS1 expression in human TNBC tumors correlates with JAK-STAT gene expression.
- Combining JAK inhibition with arginine depletion significantly suppresses tumor growth.

**What are the implications of the main findings?**

- ASS1 expression may help identify breast tumors with active cytokine/JAK-STAT signaling and refine patients’ stratification for targeted therapies.
- Inducing metabolic vulnerability through arginine depletion uncovers a targetable TME-driven survival mechanism, suggesting a new potential immunotherapeutic approach for TNBC.

**Abstract:**

Argininosuccinate synthetase 1 (ASS1) expression and arginine availability are key metabolic determinants that influence tumor fitness and regulate immune interactions within the tumor microenvironment (TME). Using an orthotopic triple-negative breast cancer (TNBC) model, we demonstrate that arginine deprivation heightens tumor dependence on the TME for survival. Mechanistically, fibroblasts sustain tumor viability by supplying arginine, whereas macrophages cooperate with stromal cues to activate Janus kinase-signal transducer and activator of transcription (JAK-STAT) signaling, thereby enhancing tumor survival. Concordantly, a JAK-STAT gene-expression signature correlates with ASS1 levels in human TNBC datasets. Translationally, combined pharmacological inhibition of JAK signaling with arginine deprivation markedly suppresses tumor growth. Together, these findings reveal a TME-driven, targetable stromal–immune circuit that enables tumors to withstand arginine deficiency-induced metabolic stress. Broadly, our work highlights that mapping and strategically inducing metabolic dependencies can reveal actionable compensatory pathways, offering opportunities to improve cancer therapy.

## 1. Introduction

The tumor microenvironment (TME) is a complex and dynamic ecosystem composed of immune, stromal, endothelial, and mesenchymal cells that collectively shape tumor growth, invasion, and responses to metabolic and therapeutic stress. Immune populations, including CD8^+^ cytotoxic and CD4^+^ helper T cells, regulatory T cells, B cells, NK cells, dendritic cells, tumor-associated macrophages, neutrophils, and myeloid-derived suppressor cells, modulate both anti- and pro-tumor immunity. In parallel, stromal components, including cancer-associated fibroblasts (CAFs), endothelial cells, and pericytes, regulate the delivery of nutrients, angiogenesis, extracellular matrix (ECM) remodeling, and the trafficking and function of immune cells. Together, these interactions strongly influence the survival and responsiveness of cancer to therapy [1,2].

Arginine metabolism intersects with these processes by shaping nutrient availability for both the tumor and the TME. Reduced extracellular arginine, driven largely by arginase-1 (ARG)-expressing tumor-associated myeloid cells, impairs CD8^+^ T cell proliferation and effector function. In macrophages, arginine fuels both inducible nitric oxide synthase (iNOS)-dependent M1 activity and ARG1-driven M2 immunosuppression. Because arginine-dependent pathways regulate tumor proliferation, nitric oxide and polyamine synthesis, angiogenesis, and ECM remodeling, therapeutic strategies that modulate arginine availability directly or via the targeting of key metabolic enzymes hold promise for reshaping the TME to enhance responses to immunotherapy [1,3].

A major determinant of tumor arginine dependence is the expression of argininosuccinate synthase 1 (ASS1), the enzyme required for endogenous arginine biosynthesis. Many cancers downregulate ASS1, rendering them arginine-auxotrophic and sensitizing them to arginine-depleting agents. Clinical evaluation of pegylated arginine deiminase (ADI-PEG20) and arginase-I has shown encouraging activity in ASS1-deficient tumors; however, re-expression of ASS1 frequently drives resistance [4,5,6,7]. We and others have further shown that ASS1 expression confers metabolic plasticity, enabling tumors to withstand nutrient stress, including glucose deprivation, and correlates with poor prognosis across several tumor types [8]. In breast cancer specifically, high ASS1 levels are associated with decreased survival [8], while ASS1 loss increases responsiveness to immune checkpoint blockade by reallocating aspartate toward pyrimidine synthesis and increasing mutational burden [9]. Thus, ASS1 expression is a critical metabolic determinant that modulates both tumor fitness and immune interactions within the TME [10].

Despite advances in immunotherapy, most patients either fail to respond or develop resistance, largely because tumors exploit metabolic and immunoregulatory circuits within the TME, underscoring the need for new and likely combined therapeutic strategies. Intensive research is being performed to uncover additional signaling nodes and cytokine pathways, including those activated under metabolic stress, that sustain immune evasion and may serve as rational combination partners for immunotherapy [11].

Based on these considerations, we hypothesized that altering arginine availability would reveal targetable TME-mediated rescue mechanisms that sustain tumor survival under arginine depletion. By uncovering a previously unrecognized mechanism through which stromal and immune cells support tumors under arginine deficiency-induced metabolic stress, our work outlines actionable TME circuits that may be leveraged for anti-cancer immunotherapy.

## 2. Materials and Methods

Detailed protocols and full methodological information are provided in the Supplementary Methods.

### 2.1. In Vivo Animal Studies

Animal experiments were approved by the Weizmann Institute IACUC (protocols 07471124-1 and 02000324-3) and conducted according to NIH, EU, and Israeli regulations.

Mice were randomly assigned to treatment groups and sample sizes, following prior publications and pilot experiments. Tumor-bearing mice were monitored daily and predefined humane endpoints were applied.

Female BALB/c mice (8–10 weeks old; Envigo, Indianapolis, IN, USA) were injected orthotopically with 0.3 × 10^6^ 4T1, 4T1-shGfp, or 4T1-sh*Ass1* cells in PBS into the lower right mammary fat pad. Mice were sacrificed between days 15 and 21 based on tumor burden; tumors were excised, weighed, and processed for downstream analyses. Terminal bleeding was performed in some experiments prior to tumor collection to analyze amino acid levels by LC-MS. For dietary arginine restriction, mice received an arginine-free diet (AFD) beginning 7–9 days prior to inoculation or starting day 11 post-inoculation, depending on the experiment. For ruxolitinib experiments, mice were size-matched on day 11 and randomized to an AFD or regular diet with oral ruxolitinib (40 mg/kg, 3×/week).

### 2.2. Flow Cytometry of 4T1 Tumors

Tumors were enzymatically digested, filtered, and processed into single-cell suspensions. Fc blocking and staining were performed using standard antibody panels for epithelial cells and myeloid subsets. Data were acquired on a CytoFLEX flow cytometer (Beckman Coulter, Brea, CA, USA) using CytExpert software (v2.6.0.105) and analyzed with FlowJo (v10.10.0). Single stains and FMOs were included in every experiment for compensation and gating.

### 2.3. NMF and BMDM Production

Primary mammary fibroblasts (NMFs) were isolated from BALB/c mammary fat pads and expanded for 6 days. Bone marrow-derived macrophages (BMDMs) were generated using L929-conditioned medium and used on day 7.

### 2.4. Cell Lines and Lentiviral Infection

4T1, GFP-4T1, and RAW264.7 cells were maintained in RPMI or DMEM supplemented with 10% FBS, glutamine, and Pen-Strep. All lines were routinely tested for Mycoplasma and maintained below passage 10. Cells were infected with pLKO-shGfp or pLKO-sh*Ass1* and selected with puromycin.

### 2.5. Arginine-Manipulated Plasmax Medium

Experiments were performed in arginine/citrulline-free Plasmax [12] with 2.5% dialyzed FBS. Arginine, citrulline, cytokines (IFNγ, IL-6, G-CSF), or ruxolitinib were supplemented as indicated.

### 2.6. Three-Dimensional (3D) Spheroid Culture and Co-Culture Systems

4T1 spheroids, 4T1-NMF co-cultures, 4T1-BMDM co-cultures, and tri-cultures were generated in Elplasia plates at defined cell ratios. Experiments were performed for 48 h under arginine-sufficient or -depleted conditions.

### 2.7. Flow Cytometry of In Vitro Samples and Apoptosis Assays

Cells or spheroids were dissociated with TrypLE (Thermo Fisher Scientific, Waltham, MA, USA) or Accumax (Innovative Cell Technologies, San Diego, CA, USA), respectively, and stained for lineage, signaling, or apoptotic markers. Annexin-V/DAPI assays were performed as per manufacturer guidelines. FMOs and single-stain controls were used for all in vitro cytometry panels.

### 2.8. LC-MS Analysis of Amino Acids

Amino acids were quantified in tumor tissue, plasma, and media using perchloric acid or solvent extraction followed by AQC derivatization.

### 2.9. XTT Survival Assay

4T1-shGfp and sh*Ass1* cells were cultured with and without arginine and citrulline, and viability was quantified by XTT kit (Biological Industries, Kibbutz Beit Haemek, Israel) according to the manufacturer’s protocol at baseline and day 4.

### 2.10. Western Blotting

Tumor and cell lysates were processed using RIPA buffer (Sigma-Aldrich, St. Louis, MO, USA), and proteins were analyzed by SDS-PAGE and immunoblotting for ASS1, JAK-STAT signaling, and immunoproteasome components. Detection used peroxidase-conjugated secondary antibodies and enhanced chemiluminescence reagents. Gels were imaged with Gel Doc XR+ (BioRad Laboratories, Hercules, CA, USA) and analyzed with ImageLab 5.1 (BioRad). Band intensities were normalized to loading controls.

### 2.11. RNA Extraction and qPCR

RNA was extracted from 3D or 2D cultures, converted to cDNA, and analyzed by SYBR-green qPCR. Primer sequences and detailed protocol are provided in the Supplementary Methods.

### 2.12. Spheroid Area Quantification

Microscopy images were acquired using an EVOS M5000 microscope (Thermo Fisher Scientific, Waltham, MA, USA) equipped with a 10× fluorite LWD objective (NA 0.30, WD 7.13 mm). Spheroid areas were quantified by manually annotating spheroid boundaries using QuPath (v0.5.1) or ImageJ (v1.54g), followed by an area calculation based on pixel measurements. For each replicate, the spheroid area in 0% arginine medium was normalized to the corresponding spheroid area in full medium, and the ratio was used for analysis.

### 2.13. Single-Cell RNA-Seq (scArg-Screen)

Tumor single-cell suspensions were enriched for viable cells and processed on the 10× Genomics Chromium platform. Libraries were sequenced on NovaSeq6000 (target ~50 k reads/cell, Illumina, San Diego, CA, USA).

### 2.14. scRNA-Seq Pre-Processing, Clustering, and Differential Expression Analysis

Raw FASTQ files were processed with CellRanger (v3.1.0) using the mm10 reference, and quality control in Seurat removed doublets, low-quality cells, and empty droplets. Samples were normalized with SCTransform, integrated, and clustered using the top 2000 variable genes, 17 principal components, and UMAP for visualization. Cell identities were assigned based on canonical marker genes. Differential gene expression between treatments was computed using the Wilcoxon Rank Sum test (log2FC ≥ 0.25, FDR ≤ 0.05), and average expression values were obtained with Seurat’s AverageExpression. Enrichment of KEGG, Reactome, and Hallmark pathways was performed using GSEA (v4.2.3) on ranked log2FC gene lists with 1000 permutations.

### 2.15. Pseudo-Bulking for Robust DGE

To avoid pseudoreplication, pseudo-bulk profiles were generated for each major cell type (>250 cells). DGE was recalculated using DESeq2 (v1.44), with FDR correction. Robustness of the pseudo-bulking strategy was confirmed by correlating results across different pseudo-bulk sizes. NK cells were excluded due to low numbers.

### 2.16. IFNγ-JAK-STAT DEG Visualization

Pseudo-bulked cancer cell data from Ctrl vs. KD + AFD groups were compared, mouse genes were mapped to human homologs using biomaRt (v2.60.1), and significant genes (FDR < 0.05, |log2FC| > 0.15) were visualized with ggplot2 (v3.5.1).

### 2.17. TCGA-TNBC Analysis

In the TNBC subset (n = 122), *ASS1* expression was correlated with IFNγ/JAK-STAT pathway genes using Spearman’s correlation. Pathway activity was computed using both mean expression and ssGSEA. *p*-values were FDR-adjusted.

### 2.18. TCGA-BRCA: ASS1-High vs. ASS1-Low

Cell-type fractions from Wang et al. were compared between *ASS1*-low and *ASS1*-high BRCA tumors using the Wilcoxon test. Receptor–ligand interactions were analyzed using LIRICS, and functional enrichment was tested by Fisher’s exact test [13].

### 2.19. Statistical Analysis

Statistical analyses were performed using ANOVA, *t*-tests, Wilcoxon tests, and FDR correction as appropriate. Significance was set at *p* < 0.05. Experiments included biological and technical replicates and were repeated independently unless stated otherwise.

Statistical tests and sample sizes for each experiment are reported in the corresponding figure legends.

## 3. Results

### 3.1. ASS1 Expression in Breast Tumors Correlates with Altered TME Composition and Signaling

To investigate whether arginine metabolism affects tumor–microenvironment interactions in breast cancer (BRCA), we first analyzed the TCGA-BRCA dataset to establish its clinical relevance. We found that breast tumors with elevated *ASS1* expression exhibited greater abundance of CAFs and immune cells compared with tumors expressing low *ASS1* (Figure S1B). Ligand–receptor interaction analysis further revealed that *ASS1* expression correlated with distinct intercellular communication patterns within the TME, involving both activating/stimulating and pro-inflammatory molecules (Figure S1C, Tables S4 and S5). These findings supported our notion that arginine metabolism in breast cancer influences both the cellular composition and signaling interactions within the TME.

### 3.2. Arginine Deprivation Strongly Impairs the Survival of 4T1 TNBC Cells In Vitro

To model these effects, we utilized the orthotopic syngeneic 4T1 mouse model of TNBC. We modulated arginine availability endogenously by altering ASS1 expression and exogenously either by removing arginine from plasma-like culture medium (Plasmax) [12] in vitro or by using an arginine-free diet (AFD) in vivo.

**Figure 1:**
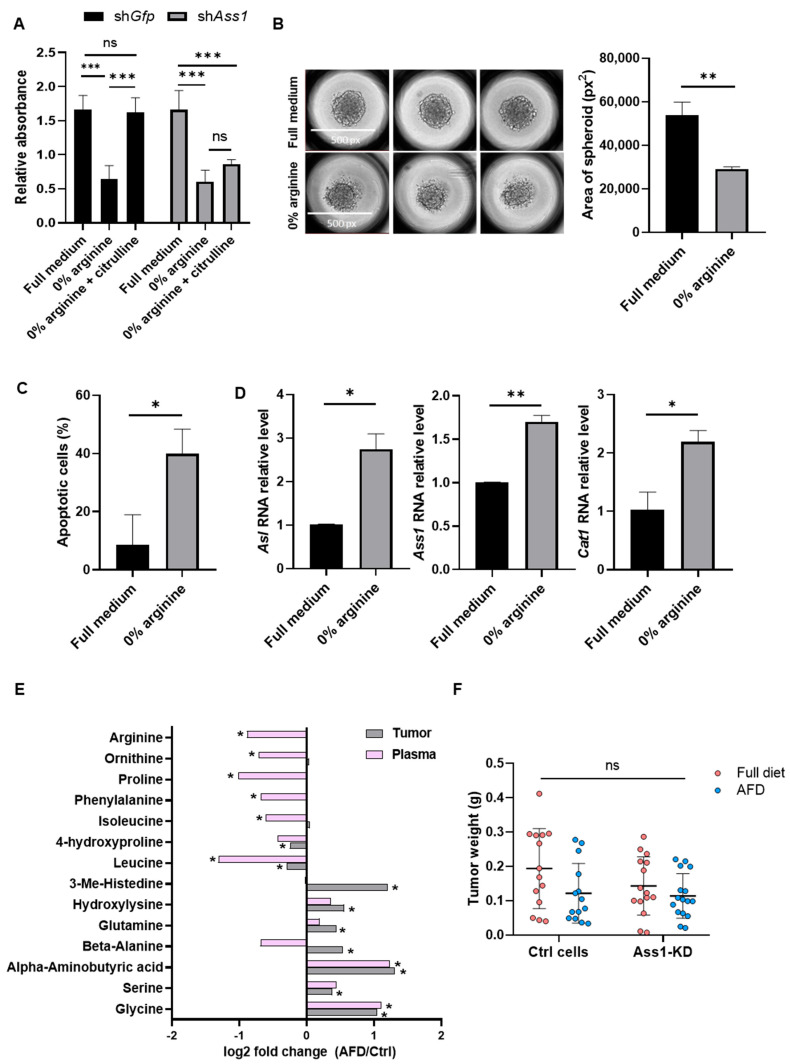
TNBC tumors resist arginine depletion despite strong in vitro sensitivity. (**A**) XTT survival assay shows reduced viability of 4T1 cells grown in arginine-deprived medium, rescued by citrulline supplementation only in ASS1-expressing cells. n = 3. Two-way ANOVA and Tukey tests. *p*-values: 0.0004, 0.0002, 0.0003, and 0.0024, respectively. (**B**) Representative images (left) and quantification (right) of 4T1 spheroid area show reduced growth in arginine-free medium. n = 3; average of 4 spheroids per replicate. Student’s *t*-test. *p*-value: 0.002. (**C**) Flow cytometry demonstrates increased apoptosis (annexin-V positive) in 3D 4T1 spheroids following arginine deprivation. n = 3. Student’s *t*-test. *p*-value: 0.015. (**D**) RT-PCR shows increased expression of *Asl* (left), *Ass1* (middle), \and *Cat1* (right) in 4T1 cells under arginine deprivation. n = 2. Student’s *t*-test. *p*-values: 0.020, 0.005, and 0.012, respectively. (**E**) LC-MS shows significant changes in amino acid levels in plasma and tumors of mice fed with the AFD vs. the standard diet. Values are presented as log2 fold change. n = 13–14 per group; 3 experiments. Two-way ANOVA and Tukey tests. *p* value < 0.05. (**F**) Tumor weights of BALB/c mice injected with 4T1-sh*Gfp* or 4T1-sh*Ass1* and fed with a standard diet or an AFD demonstrate limited reduction following in vivo arginine deprivation. n = 14–16 per group; 3 experiments. Two-way ANOVA and Tukey tests. ns—not significant, * *p* < 0.05, ** *p* < 0.01, and *** *p* < 0.001.

We first validated the essentiality of arginine for 4T1 cells by culturing ASS1-downregulated and -expressing cells in an arginine-depleted Plasmax medium. We found that arginine depletion reduced 4T1 cell survival, while supplementation with the ASS1 substrate citrulline rescued survival only in ASS1-expressing 4T1 cells, confirming their reliance on ASS1 for arginine synthesis and on arginine for survival (Figure 1A, and Figure S1A and S2A). We further validated the essential role of arginine in 4T1 survival by showing restricted growth and increased apoptosis in 3D spheroids cultured in arginine-depleted Plasmax medium (Figure 1B,C). As expected, arginine deprivation induced compensatory responses in 4T1 cells, including increased expression of *Ass1*, *Asl*, and the *cationic amino acid transporter 1 (Cat1)*, a key arginine transporter (Figure 1D) [14]. However, these compensatory mechanisms were insufficient to sustain cell survival.

### 3.3. 4T1 TNBC Tumors Resist Arginine Depletion In Vivo

We next examined the effects of systemic arginine restriction in vivo. Orthotopic injection of 4T1 cells into the mammary fat pads of mice fed with an AFD resulted in reduced levels of arginine and several other amino acids in plasma, while elevating amino acid levels within tumors, compared to control mice fed with a normal diet (Figure 1E). These results indicate that arginine deprivation induces systemic metabolic shifts and highlight the ability of tumors to exploit host resources under nutrient stress.

To further limit arginine supply, we combined systemic arginine depletion with tumor-intrinsic suppression of de novo synthesis by implanting 4T1 cells with knockdown (KD) of ASS1(ASS1-KD) into AFD-fed mice (Figure S2B). Surprisingly, despite the strong in vitro dependency of cancer cells on arginine for survival, inhibition of both endogenous and exogenous arginine supply failed to significantly impair tumor growth in vivo (Figure 1F), suggesting the presence of protective mechanisms mediated by components of the TME.

### 3.4. Arginine Deprivation Triggers Stress Responses and Broad Transcriptional Changes in Cancer Cells In Vivo

To identify TME-driven compensatory responses to arginine depletion, we performed a single-cell RNA-sequencing screen (scArg-screen) of cancer, stromal, and immune cells within 4T1 tumors subjected to different modes of arginine modulation (Figure S2B,C).

**Figure 2:**
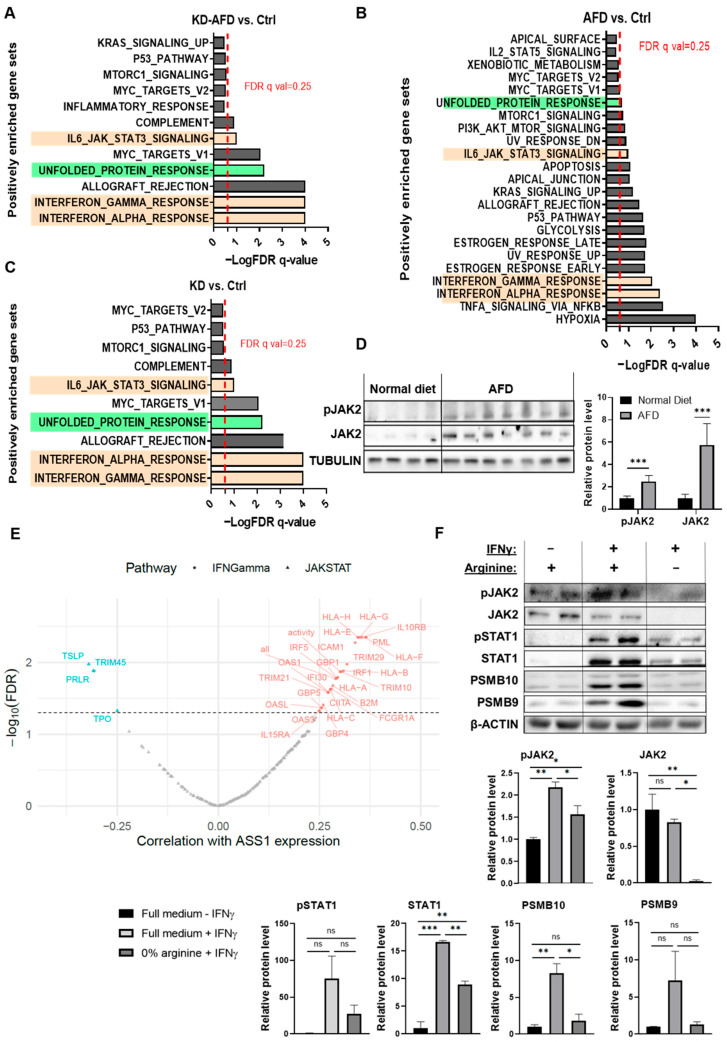
Arginine deprivation activates IFNγ/JAK-STAT in vivo despite in vitro suppression. (**A**–**C**) Pathway enrichment of Hallmark gene sets of KD + AFD vs. Ctrl (**A**), AFD vs. Ctrl (**B**), and KD vs. Ctrl. (**C**) Shows upregulation of unfolded protein response (green) and IFNγ/JAK-STAT signaling (light orange) in cancer cells from the single-cell RNA sequencing following different modes of arginine restriction (scArg-screen). BALB/c female mice were inoculated with 4T1-sh*Gfp* or 4T1-sh*Ass1* cells and fed with a standard diet or an AFD. Tumors of 4–5 mice from each group were pooled together. Red line = FDR q-value threshold of 0.25. (**D**) Western blot (left) and its quantification (right) of pJAK2 and JAK2 expression in tumors from mice fed with a standard diet (n = 4) or an AFD (n = 7) on day 7 after tumor inoculation. Student’s *t*-test. *** *p*-value < 0.001. (**E**) Spearman correlations in TCGA-TNBC tumors show a correlation between *ASS1* and IFNγ/JAK-STAT genes. Red = positive correlation; blue = negative correlation. Significant genes (FDR < 0.05) are colored; “all” and “activity” reflect gene average and estimated activity by single-sample gene set enrichment analysis (ssGSEA), respectively. Full data are provided in Table S2. (**F**) Western blot (upper) and its quantification (lower) show IFNγ-induced JAK-STAT-PSMB9-10 expression in 4T1 spheroids only in the presence of arginine. n = 2 replicates. The experiment was repeated 3 times. Student’s *t*-test. ns—not significant, * *p* < 0.05; ** *p* < 0.01; *** *p* < 0.001.

By first focusing on the cancer cells within the tumor, we confirmed that *Ass1* expression levels were reduced in cancer cells of the KD and KD + AFD groups and elevated in the AFD group (Figure S2D). Notably, gene set enrichment analysis (GSEA) revealed an increase in the unfolded protein response in cancer cells across all groups, regardless of the modality used to reduce arginine levels, indicating induction of cellular stress (Figure 2A–C). In addition, similar pathway alterations were observed in cancer cells following either ASS1 knockdown or an AFD, underscoring the need for coordinated metabolic adaptation to both cell-intrinsic and systemic arginine deficiency (Figure S2E and Table S1). These findings further underscore the crucial role of arginine in supporting breast cancer cell survival and the programmed responses triggered upon its depletion.

### 3.5. Arginine Starvation Upregulates IFNγ-JAK-STAT Signaling in 4T1 Tumors

Because our a priori hypothesis was that arginine deprivation would induce tumor-microenvironment-dependent transcriptional responses in cancer cells, we next examined which pathways were consistently altered across all three modes of arginine starvation. This analysis highlighted the interferon-Janus kinase-signal transducer and activator of transcription (JAK-STAT) axis as the most recurrently and significantly enriched pathway, aligning with the expected TME-driven response pattern. The main enriched pathways included interferon-alpha (IFN) and gamma (IFNγ) signaling, as well as, to a lesser extent, IL6-JAK-STAT3 signaling (Figure 2A–C, Tables S1 and S6). Interferon signaling is mediated by activation of Janus kinase 2 (JAK2) [15]. Indeed, Western blot analysis of 4T1 tumors following an AFD confirmed the elevation in JAK2 phosphorylation and total protein levels (Figure 2D). In concordance with the elevation in IFNγ response following depletion of arginine, further analysis of the cancer cells’ scArg-screen data revealed an elevation in canonical downstream targets of IFNγ-JAK2-STAT1, including genes of the immunoproteasome (IP) subunits and genes related to antigen presentation (Figure S3A–C). Accordingly, we further examined the expression of key components of these IFN-γ-regulated pathways, including major histocompatibility complex class I (MHC-I) and the IP subunits proteasome subunit beta type-9 (PSMB9/LMP2) and proteasome subunit beta type-10 (PSMB10/MECL-1), as functional readouts of IFNγ pathway activation [16,17]. Specifically, we confirmed by flow cytometry analysis the elevation in MHC-I expression in ASS1-KD + AFD tumors (Figure S3D).

Notably, in support of the human relevance of our findings for TNBC patients, analysis of TCGA-TNBC data showed a correlation between *ASS1* expression levels and the expression of IFNγ and JAK-STAT-associated genes (Figure 2E and Table S2). Importantly, these correlations encompassed both positive and negative associations, suggesting that *ASS1* levels are correlated with the overall transcriptional activity of the IFNγ-JAK-STAT axis, rather than with a single directional change. The presence of coordinated regulation between ASS1 and multiple cytokine response genes in human tumors aligns with our experimental findings and further supports the conserved connection between arginine metabolism and inflammatory signaling in TNBC.

### 3.6. 4T1 Cancer Cells Show Suppressed JAK-STAT Signaling Under Arginine Depletion

In contrast to these in vivo results, we found that adding IFNγ to 4T1 spheroids cultured under arginine deprivation inhibited the induction of the downstream JAK2 and STAT1 proteins and their phosphorylated forms, consequently decreasing the expression of IFNγ-regulated genes MHC-I and PSMB9 (Figure 2F and Figure S4A,B). Moreover, the addition of citrulline could restore the levels of total and phosphorylated STAT1 and PSMB9 proteins in response to IFNγ in ASS1-expressing cancer cells grown in an arginine-depleted medium, confirming the essentiality of arginine for the cancer cell response to IFNγ (Figure S4C). Notably, we found that the in vitro inhibition of JAK2 signaling following arginine depletion was not specific to IFNγ, as the addition of Interleukin-6 (IL-6) or Granulocyte colony-stimulating factor (G-CSF) cytokines, known activators of JAK signaling [18], led to a similar decrease in JAK2 phosphorylation (Figure S4D).

The contrasting responses to arginine depletion, demonstrating enhanced IFNγ-JAK-STAT activation with limited tumor growth inhibition in vivo, versus suppressed IFNγ signaling and heightened sensitivity in vitro, suggest that IFNγ signaling in cancer cells may be a compensatory rescue mechanism induced by the cross-talk between cancer cells and other cells in the TME to support tumor cell survival under arginine-deprived conditions.

### 3.7. Stromal and Immune Cells Exhibit Distinct Transcriptional Responses to Arginine Depletion

**Figure 3:**
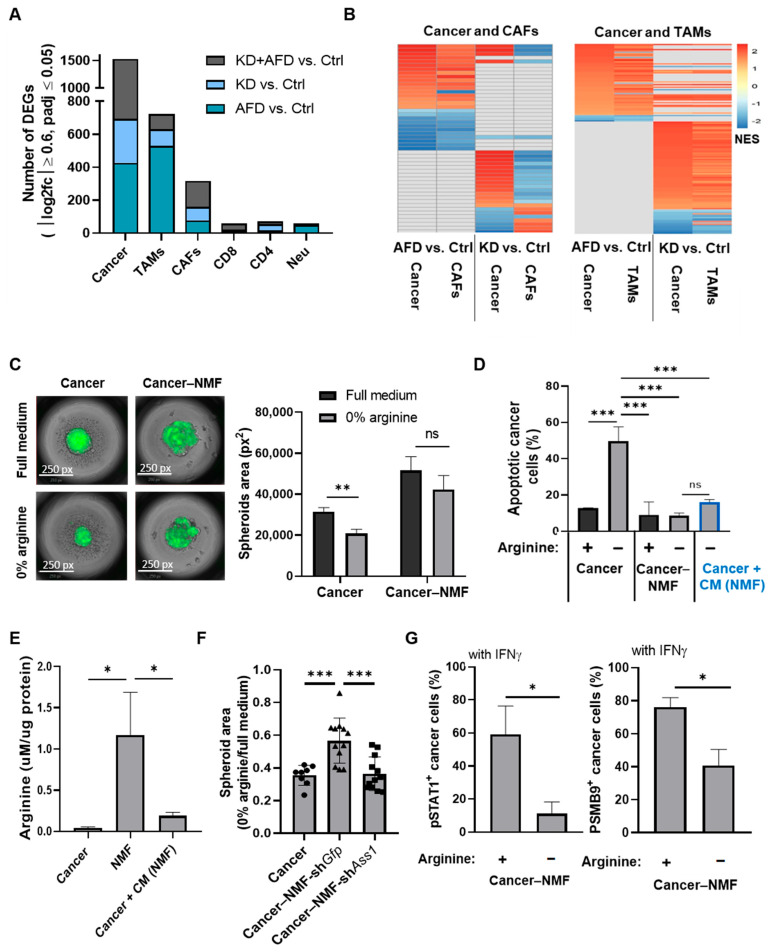
CAF-derived arginine supports cancer cell survival but does not rescue IFNγ-JAK-STAT signaling. (**A**) Bar graph shows the number of DEGs in each TME cell type from the scArg-screen, indicating similar transcriptomic effects following cancer-endogenous and systemic inhibition of arginine availability. DEGs were determined using 12 pseudo-samples per treatment as described in the Methods Section. (**B**) Heatmaps show GSEA-enriched pathways (*p* ≤ 0.05) shared between cancer cells and CAFs (left) or cancer and TAMs (right), based on the scArg-screen. In each graph, the pathways shown in the 2 columns on the left compare AFD vs. Ctrl, and the 2 columns on the right compare KD vs. Ctrl. Full data is provided in Table S3. (**C**) Representative images (left) and quantification (right) of GFP+ 4T1 cells co-cultured with NMFs demonstrate preserved spheroid size in arginine-deprived conditions. n = 3. Four spheroids were averaged for each replicate. Student’s *t*-test. *p*-value: 0.003. (**D**) Flow cytometry of annexin-V staining shows reduced apoptosis in 4T1 cells co-cultured with NMFs or treated with conditioned medium of NMFs. n = 3. One-way ANOVA and Tukey tests. *** *p*-value < 0.001. (**E**) LC-MS analysis shows elevated arginine levels in NMF medium compared to cancer cell medium or to medium of cancer cells treated with CM from NMF. Values were normalized to total protein mass. n = 3; one-way ANOVA and Tukey tests. *p*-values: 0.29 and 0.32, respectively. (**F**) Quantification of spheroids’ area of 4T1 monoculture (4T1) or 4T1 co-cultured with NMFs expressing shGfp (NMF-sh*Gfp*) or sh*Ass1* (NMF-sh*Ass1*). Preserved spheroid growth under arginine-deprived conditions was observed only when 4T1 cells were co-cultured with NMFs expressing *Ass1*. (n = 3; four spheroids analyzed per replicate. One-way ANOVA and Tukey tests. *** *p*-value < 0.001. (**G**) Flow cytometry shows reduced pSTAT1^+^ (left) and PSMB9^+^ (right) cancer cells co-cultured with NMFs under arginine deprivation, indicating fibroblasts do not rescue IFNγ signaling under arginine deprivation. n = 3. Student’s *t*-test. *p*-values: 0.011 and 0.005, respectively. ns—not significant, * *p* < 0.05; ** *p* < 0.01; *** *p* < 0.001.

Focusing on the cellular components of the TME, analysis of the scArg-screen revealed that both systemic and intrinsic arginine restriction induced substantial transcriptional changes in stromal and immune cells, particularly in TAMs and CAFs (Figure 3A and Figure S5A–F). GSEA further showed that pathway responses to arginine depletion differed between CAFs and TAMs. Under systemic restriction by an AFD, most shared pathways in both cell types responded similarly to tumor cells. However, in contrast, endogenous inhibition of arginine synthesis in tumor cells through ASS1 knockdown revealed a distinct divergence in response: whereas tumor cells and TAMs displayed similar overall trends in the direction of transcriptional changes, tumor cells and CAFs exhibited opposing patterns (Figure 3B). Notably, the shared pathways between tumor and TAMs included elevated interferon signaling (Table S3). These results indicate shared dependencies between tumor cells and TAMs following systemic and cancer-specific arginine depletion, suggesting a potential compensatory mechanism in CAFs that enables cancer survival.

### 3.8. Fibroblast-Derived Arginine Supports Cancer Cell Survival but Does Not Trigger Elevated IFNγ-JAK-STAT Signaling Following Arginine Deprivation

To better differentiate the contributions of CAFs and TAMs to the response of cancer cells to arginine deprivation, we first established a co-culture system of 4T1 cancer cells and normal mammary fibroblasts (NMFs). Although not tumor-derived, NMFs are influenced by breast tumor cells in co-culture, acquiring CAF-like gene expression and functional features [19,20]. Interestingly, a 3D co-culture of 4T1 and NMFs revealed that NMFs provide cancer cells with resistance to arginine deficiency, as can be seen by the preservation of the size of the spheroids and significant reduction in apoptosis in cancer cells grown in co-culture compared to cancer cells grown without NMFs (Figure 3C,D). Notably, conditioned medium (CM) from NMFs cultured in an arginine- and citrulline-depleted medium provided cancer cells with similar protection against the apoptosis induced by arginine deficiency, supporting the notion that fibroblasts secreted molecules protect cancer cells against arginine deficiency (Figure 3D). Indeed, arginine levels were elevated in the medium of the NMFs cultured in arginine- and citrulline-free medium, while applying this medium on cancer cells decreased arginine levels, likely due to arginine consumption by cancer cells (Figure 3E). To validate the role of NMFs’ secreted arginine in rescuing the cancer cells’ survival under arginine deprivation, we silenced the expression of ASS1 in NMFs co-cultured with cancer cells. Indeed, the silencing of ASS1 in NMFs abolished the rescue of the cancer cells’ survival by fibroblasts (Figure 3F and Figure S6A,B).

However, although NMFs secreted arginine rescued cancer cells from apoptosis under arginine-deprived conditions, co-culture with NMFs alone failed to restore JAK-STAT signaling in cancer cells exposed to IFNγ, as indicated by persistently low pSTAT1 and PSMB9 protein levels (Figure 3G). These findings suggest that arginine supplementation by primary fibroblasts is insufficient to recapitulate the full IFNγ-JAK-STAT activation observed in vivo. Thus, we next co-cultured 4T1 cells with RAW 264.7 cells and primary bone marrow-derived macrophages (BMDMs), which, similar to NMFs, are not tumor-derived but become reprogrammed by breast cancer cells in co-culture to acquire TAM-like phenotypes [21,22].

### 3.9. Cooperative Cross-Talk Between Cancer Cells, Fibroblasts, and Macrophages Mediates JAK-STAT Activation Under Arginine Deprivation

**Figure 4:**
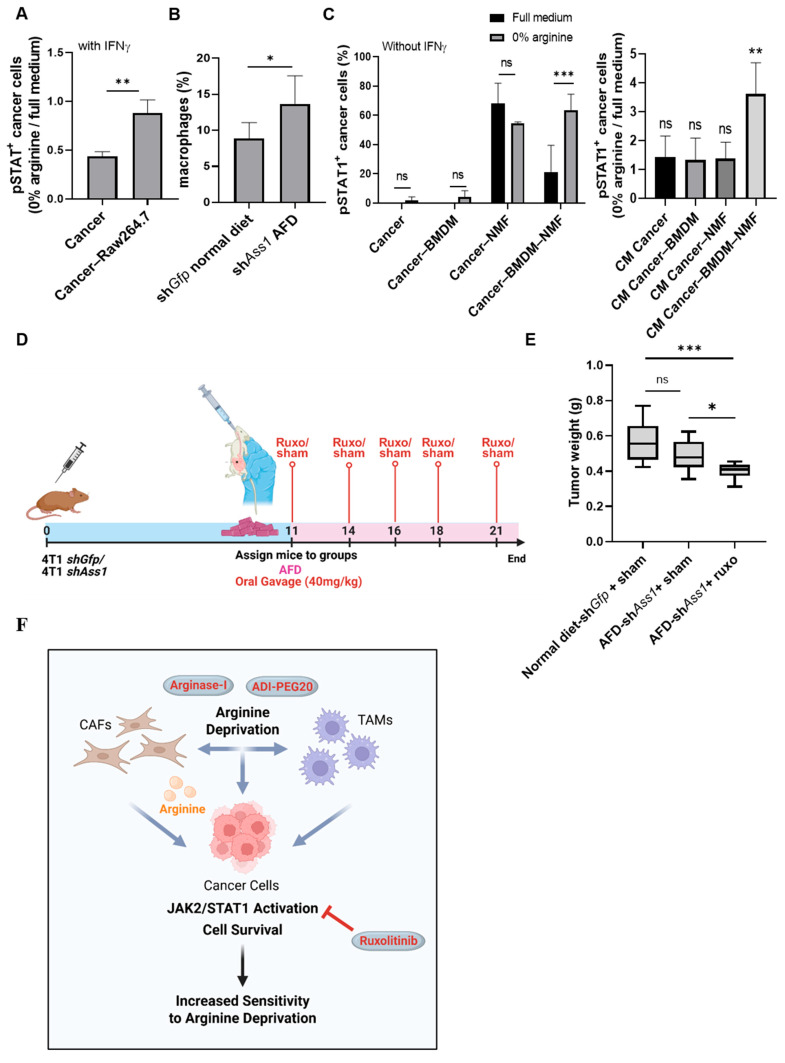
Cancer–CAF–TAM crosstalk drives JAK-STAT activation under arginine deprivation, revealing therapeutic vulnerability in TNBC. (**A**) Flow cytometry shows elevated IFNγ-induced pSTAT1^+^ cancer cells (0% arginine/full medium) when co-cultured with RAW264.7 macrophages. n = 3. Student’s *t*-test. *p*-value: 0.006. (**B**) Flow cytometry shows increased CD45^+^CD11b^+^F4/80^+^ macrophages in tumors of KD + AFD mice vs. Ctrl. n = 5. Student’s *t*-test. *p*-value: 0.043. (**C**) Flow cytometry analysis of pSTAT1^+^ cancer cells shows increased pSTAT1 activation under arginine deprivation when 4T1 cells are co-cultured simultaneously with BMDMs and NMFs (left) or when treated with CM derived from this tri-culture (right). No external IFNγ was added. For each experiment: n = 3, 2-way ANOVA with Tukey’s tests comparing full vs. 0% arginine within each co-culture/CM condition (*p* = 0.001 and *p* = 0.0054, respectively). (**D**) Schematic overview of experimental design: BALB/c female mice were injected with 4T1-shGfp or 4T1-sh*Ass1* (ASS1-KD) cells. On day 11, mice were divided into 2 groups: mice fed with a regular or arginine-free diet (AFD) and treated by gavage with ruxolitinib (40 mg/kg). Created in Biorender. Tishler, H. (2025) https://BioRender.com/v7fnfh3. (**E**) Tumor weights show enhanced response to arginine deprivation when mice are treated with ruxolitinib. n = 6–7. One-way ANOVA test. *p*-value < 0.0001 and 0.021, respectively. (**F**) Proposed model: Arginine depletion in tumor cells elicits a compensatory response mediated by the tumor microenvironment. CAFs support cancer cells by supplying arginine, and cooperative interplay among cancer cells, CAFs, and TAMs activate JAK-STAT signaling to sustain cancer cell survival. This acquired dependency sensitizes tumors to JAK1/2 inhibition with ruxolitinib, offering a strategy to overcome resistance to arginine-depleting agents such as ADI-PEG20 and Arginase-I. Created in Biorender. Tishler, H. (2025) https://BioRender.com/cyfg4aa. ns—not significant, * *p* < 0.05; ** *p* < 0.01; *** *p* < 0.001.

Encouragingly, co-culturing of 4T1 cells with RAW 264.7 macrophage cells in an arginine-depleted medium supplemented with IFNγ significantly increased pSTAT1 levels in cancer cells relative to culturing in full medium, supporting a role for macrophages in enabling the induction of JAK-STAT signaling in tumor cells under arginine depletion (Figure 4A). In line with these findings, and with the increased prevalence of macrophages in TCGA breast cancer tumors expressing high levels of ASS1, in vivo arginine depletion increased macrophage abundance in the TME of 4T1 tumors (Figure S1B and Figure 4B). To better mimic the TME, we established a tri-culture system comprising cancer cells, BMDMs, and NMFs, without exogenous IFNγ supplementation. Spheroid size was preserved following co-cultures with NMFs, BMDMs, or both under arginine deprivation (Figure S6C). However, strikingly, we found that exclusively in the tri-culture setting, arginine deprivation induced elevated STAT1 phosphorylation, resembling the in vivo response (Figure 4C). Moreover, conditioned medium from arginine-deprived tri-cultures, but not from cancer cell and NMF or cancer cell and BMDM co-cultures, was sufficient to increase pSTAT1 levels in cancer cells. Collectively, these results demonstrate that arginine depletion promotes cooperative crosstalk between cancer cells, fibroblasts, and macrophages, driving STAT1 activation to support tumor survival.

### 3.10. Combined Arginine Deprivation and JAK Inhibition Reveals a Therapeutically Targetable Vulnerability in TNBC

The unique activation of IFNγ-JAK-STAT signaling in cancer cells following arginine deprivation prompted us to examine the potential therapeutic effect of the JAK1/2 inhibitor ruxolitinib [23]. Importantly, combining arginine-intrinsic (ASS1-KD) and -extrinsic (AFD) deprivation with ruxolitinib gavage treatments in 4T1-bearing mice, starting on day 11 when 4T1 tumors are palpable, significantly reduced tumor size (Figure 4D,E).

These findings demonstrate that arginine deprivation sensitizes TNBC to JAK signaling inhibition, revealing a targetable therapeutic vulnerability.

## 4. Discussion

Our findings reveal that arginine deprivation activates a TME-driven JAK-STAT rescue pathway in TNBC, defining a biologically coherent and therapeutically actionable dependency. Although 4T1 TNBC cells were highly sensitive to arginine depletion in vitro, combined inhibition of endogenous arginine synthesis and systemic depletion in vivo yielded only modest reductions in tumor growth. This discrepancy highlights a key finding of our study: the TME serves as a compensatory support that buffers tumors against nutrient stress. Single-cell transcriptomic profiling uncovered coordinated transcriptional remodeling across stromal and immune compartments, identifying CAFs and TAMs as key mediators of this adaptive response. Our data supports a model in which fibroblast-derived arginine maintains tumor viability under arginine deprivation, while coordinated CAF–macrophage signaling activates the JAK-STAT signaling pathway. In this way, arginine depletion exposes a JAK-STAT rescue circuit, wherein stromal–immune cooperation reinstates pro-survival transcriptional programs under metabolic stress. Consistent with this model, pharmacologic JAK inhibition effectively disrupted the adaptive response and enhanced the anti-tumor efficacy of arginine depletion, validating JAK-STAT as a strategic node for metabolic–immunologic combination therapy.

Our analysis of TCGA-BRCA and TCGA-TNBC cohorts further supports the human relevance of our findings. Tumors with higher *ASS1* expression displayed an increased abundance of fibroblasts and macrophages, as well as a coordinated upregulation of IFN-γ- and JAK-STAT-associated genes, mirroring the metabolic–immune interactions uncovered in our mouse model. These observations suggest that *ASS1* expression marks broader TME remodeling involving JAK -STAT activation, reinforcing the translational significance of the arginine–JAK-STAT axis described here for therapy.

This mechanism aligns with broader evidence that dysregulated JAK-STAT signaling is a convergent axis of treatment resistance across multiple cancers, including TNBC and hepatocellular carcinoma (HCC). In TNBC, constitutive or stress-induced activation of JAK2-STAT3 and, increasingly appreciated, STAT1 promotes proliferation, invasion, stemness, and immune evasion, correlating with chemotherapy failure and poor prognosis [24,25,26,27]. Similarly, in HCC, resistance to sorafenib and other targeted therapies arises through the coordinated rewiring of pathways, such as the PI3K-AKT and JAK-STAT pathways, within a hypoxic, fibroblast-rich microenvironment that fosters epithelial–mesenchymal transition, cancer stemness, and immune escape. Mechanistic and bioinformatic studies have positioned JAK-STAT signaling at the center of this adaptive remodeling [28]. Notably, JAK-STAT-dependent transcriptional signatures can display features of immune activation while simultaneously encoding immune-evasive states, illustrating a functional duality that likely extends to metabolically stressed TNBC [29].

Our results further refine the emerging view of STAT1 as a context-dependent mediator of tumor progression. Although STAT1 is classically associated with tumor-suppressive interferon responses, mounting evidence suggests that in TNBC, STAT1 can acquire pro-tumorigenic functions, promoting stemness, proliferation, inflammatory cytokine production, and poor clinical outcomes [24,30]. Indeed, in our model, STAT1 upregulation following arginine deprivation appears to reflect a stress-induced adaptive state that enhances tumor resilience. This dual functionality highlights the importance of targeting pathway-level vulnerabilities rather than individual cytokines or transcription factors, especially in tumors that exhibit rapid metabolic and transcriptional plasticity.

From a therapeutic perspective, our data suggest that ASS1-low tumors, which cannot compensate for the loss of extracellular arginine, are particularly vulnerable to arginine deprivation, whereas ASS1-high tumors exhibit reduced dependence on exogenous arginine and display a stronger IFNγ-JAK-STAT signature. Thus, ASS1 expression levels may help distinguish tumors with intrinsic metabolic dependency from those buffered by their microenvironment and could serve as a basis for patient stratification.

Broadly, the field of cancer metabolism has been driven by the idea that depriving tumors of a specific nutrient exposes a vulnerability that can be exploited with targeted drugs. Here, we demonstrate that a deeper understanding of tumor metabolism, particularly its dynamic crosstalk with the TME, reveals not only how cancer cells adapt to metabolic stress, but also how stromal and immune cells co-evolve to sustain tumor growth. In the context of arginine starvation in TNBC, this reciprocal interaction triggers the JAK-STAT compensatory responses, which can be targeted for therapeutic intervention. Hence, by pinpointing the nodes where tumor and TME dependencies converge to enhance survival, we uncover therapeutic windows in which existing drugs can effectively block these adaptive pathways. This strategy reframes metabolic plasticity: rather than a barrier to therapy, it becomes an opportunity to achieve more durable and effective treatments.

### Limitations and Future Directions

While our findings propose a compelling biological model, several limitations warrant consideration. First, we have not yet defined the specific factors and interactions among CAFs, TAMs, and cancer cells that ultimately generate the microenvironmental signals driving JAK–STAT activation under arginine deprivation. Targeted perturbation of IFNγ, IL-6, and other candidate pathways will be required to establish causality. Second, although JAK inhibition enhanced the efficacy of arginine depletion, validation across additional TNBC models, including patient-derived systems, will be necessary to determine the generalizability of these therapeutic effects. Finally, we did not assess compensatory pathways that may emerge after dual metabolic and signaling inhibition. Identifying such secondary escape routes will be essential for designing durable, mechanism-based therapeutic combinations.

## 5. Conclusions

Collectively, our study demonstrates that arginine deprivation reveals a TME-driven JAK-STAT rescue circuit that enables TNBC tumors to withstand metabolic stress, uncovering a coordinated survival program that spans both the stromal and immune compartments. By integrating metabolic, transcriptomic, and in vivo analyses, we demonstrate that CAF-derived arginine supports tumor cell viability under deprivation, while the full activation of JAK-STAT signaling in the tumor and the subsequent survival despite systemic nutrient depletion requires the combined contribution of stromal cells and macrophages. This multifaceted adaptive response underscores the TME’s central role in buffering metabolic therapies and identifies JAK-STAT activation as a critical convergence point through which stromal–immune interactions reestablish tumor fitness. Importantly, pharmacologic JAK inhibition disrupts this rescue mechanism, significantly enhancing the anti-tumor efficacy of arginine deprivation and defining a tractable therapeutic strategy for otherwise refractory TNBC. These findings advance a mechanistic rationale for combining metabolic stress with targeted disruption of adaptive immune signaling pathways, reframing metabolic plasticity as a source of exploitable vulnerability and offering a clear translational path toward more durable, mechanism-based treatment regimens in TNBC.

## Data Availability

All data are available in the main text or the Supplementary Materials. The data of the single-cell RNA sequencing screen (scArg-screen) has been deposited in GEO under accession number GSE310301.

## Supplementary Materials

The following supporting information can be downloaded at: https://www.mdpi.com/article/10.3390/cells15010025/s1, Figure S1: *ASS1* levels in TCGA-BRCA samples correlate with immune cell infiltration and intercellular interactions; Figure S2: Validation of *Ass1* knockdown and the scArg-screen; Figure S3: Increased in vivo IFNγ/JAK-STAT signaling; Figure S4: Arginine deprivation suppresses IFNγ signaling in vitro; Figure S5: Cell-type-specific transcriptional responses to arginine deprivation; Figure S6: Fibroblast- and macrophage-mediated support preserves cancer cell survival under arginine deprivation; Table S1: Shared GSEA-enriched pathways (*p* ≤ 0.05) in KD vs. Ctrl and AFD vs. Ctrl cancer cells from 4t1 tumors of the scArg-screen. NES—normalized enriched score; Table S2: Correlation of *ASS1* expression with IFNγ/JAK–STAT pathway genes and activity. “All” indicates the average expression of all pathway genes, and “activity” reflects pathway activity estimated by ssGSEA; Table S3: GSEA-enriched pathways (*p* ≤ 0.05) shared between cancer cells and CAFs or cancer cells and TAMs, based on the scArg-screen; Table S4: Enriched ligand-receptor interactions of high-*ASS1* tumors, based on TCGA breast cancer samples; Table S5: Enriched ligand-receptor interactions of low-*ASS1* tumors, based on TCGA breast cancer samples; Table S6: Consistently enriched Hallmark pathways across KD, KD-AFD and AFD vs. control comparisons, with roles in cancer cell adaptive survival and TNBC-specific evidence; Supplementary Methods and Western blots. References [31,32,33,34,35] are cited in the supplementary materials.
