# Supplementary material for: Inhibition of Tumor Microenvironment-Driven JAK-STAT Signaling Enhances Response to Arginine Deprivation Therapy in Triple-Negative Breast Cancer
Source: Cells. 2025 Dec 23;15(1):25. doi: 10.3390/cells15010025 (PMC12785028; doi:10.3390/cells15010025)
Supplement: Supplementary file 1 [file cells-15-00025-s001.zip › Supp cells-4016445 proof/Cells-4016445 Supplementary Materials.pdf]

Supplementary Materials for

Inhibition of Tumor Microenvironment-Driven JAK-STAT Signaling Enhances Response to Arginine Deprivation Therapy in Triple-Negative Breast Cancer

Hila Tishler *et al.*

*Corresponding author ayelet.erez@weizmann.ac.il

This PDF file includes:

Figures S1 to S6
Table S6
Legends for Supplementary Tables S1–S5 (Excel)
Legend for the Western blot source file (PDF)

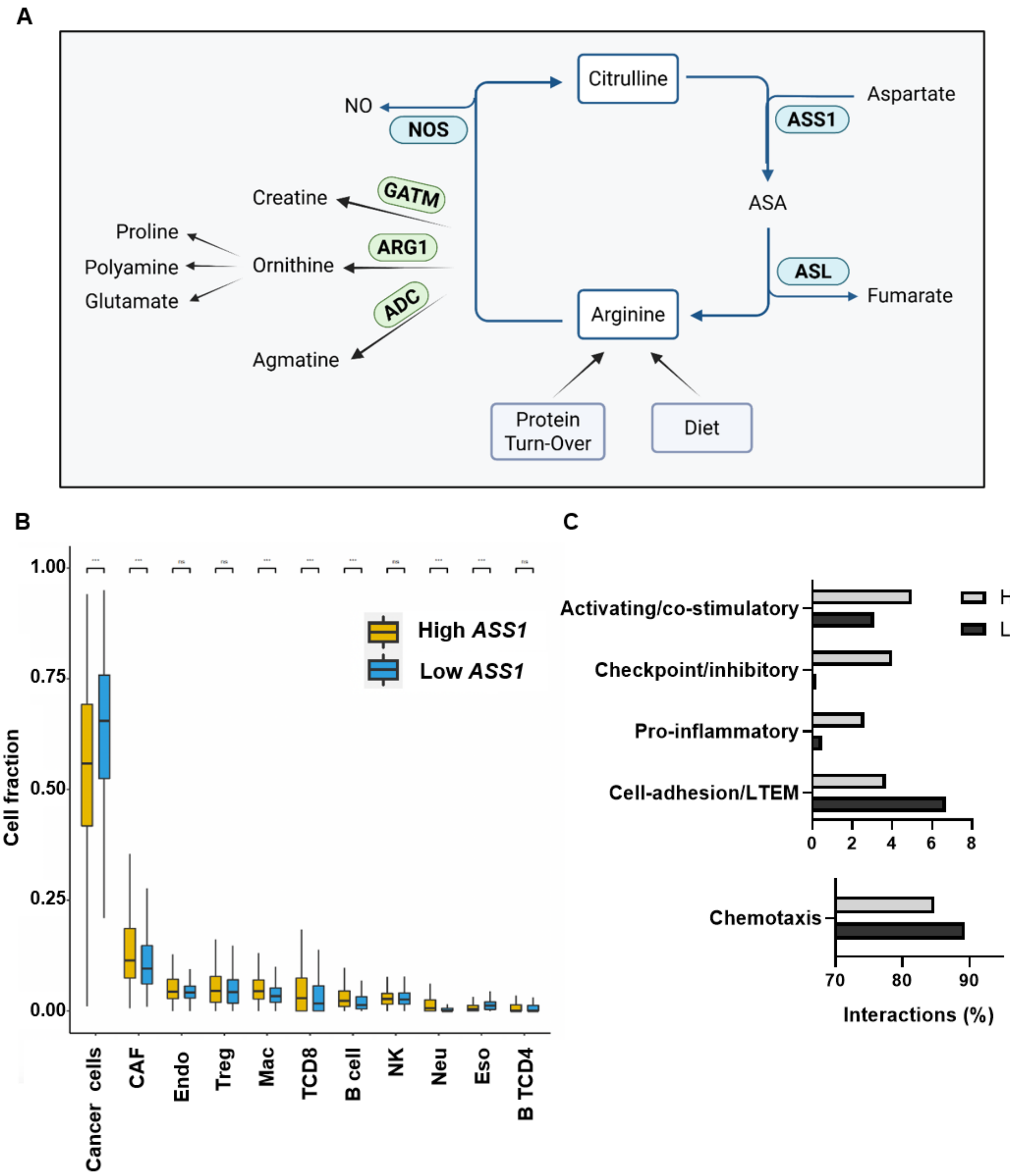

Figure S1. ***ASS1*** **levels in TCGA-BRCA samples correlate with immune cell infiltration and intercellular interactions**

(A) Scheme of arginine metabolism. Arginine is acquired via dietary intake, protein breakdown, or endogenous synthesis through the citrulline-NO cycle. It serves as a substrate for four enzymes producing NO, polyamines, glutamate, and creatine. Created in BioRender. Erez, A. (2025) https://BioRender.com/iqy4e6b **(B)** Boxplot showing abundance of various TME cell types in TCGA breast cancer samples with high vs. low *ASS1* expression, showing increased immune infiltration in high-*ASS1* tumors. ***p-value<0.001.
(C) Ligand-receptor interaction network based on TCGA breast cancer samples. Low-*ASS1* tumors show enrichment of chemotaxis and cell-adhesion/LTEM signaling interactions in comparison to the high-*ASS1* group. High-*ASS1* tumors demonstrate enrichment of checkpoint/inhibitory and pro-inflammatory interactions. Full data is provided in **Table S4** (high-*ASS1* tumors) and **Table S5** (low-*ASS1* tumors).

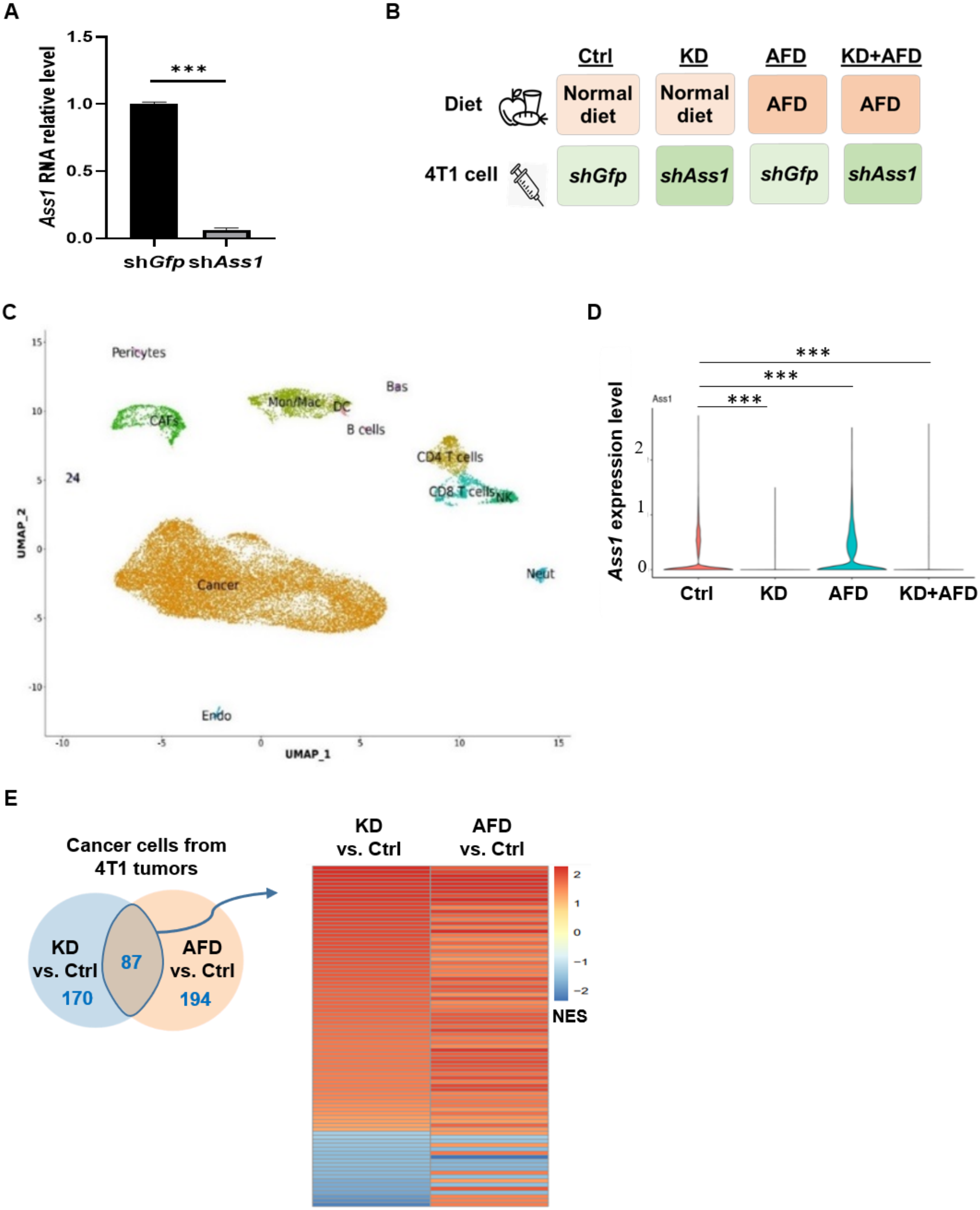
A
Ass1 RNA relative level
1.5
1.0
0.5
0.0
***
shGfp
shAss1
B
Ctrl
KD
AFD
KD+AFD
Diet
Normal diet
Normal diet
AFD
AFD
4T1 cell
shGfp
shAss1
shGfp
shAss1
C
Pericytes
Bas
Mon/Mac
DC
CAFs
B cells
CD4 T cells
CD8 T cells
NK
24
Cancer
Neut
Endo
UMAP_1
UMAP_2
D
Ass1 expression level
***
***
***
Ctrl
KD
AFD
KD+AFD
E
Cancer cells from 4T1 tumors
KD vs. Ctrl
170
87
AFD vs. Ctrl
194
KD vs. Ctrl
AFD vs. Ctrl
NES

Figure S2. Validation of *Ass1* knockdown and the scArg-screen

(A) RT-PCR analysis shows reduced *Ass1* expression in 4T1 cells following sh*Ass1* knockdown. n=2. Student's t-test. p-value: 0.0001.

(B) Schematic description of arginine restriction experimental treatment groups: Ctrl (sh*Gfp* + regular diet), KD (sh*Ass1* + regular diet), AFD (sh*Gfp* + AFD), KD+AFD (sh*Ass1* + AFD).

(C) Uniform Manifold Approximation and Projection for Dimension Reduction (UMAP) plot of the scArg-screen showing manual cell type annotation, identified by marker expression.

(D) Violin plots based on the scArg-screen show *Ass1* expression in cancer cells, demonstrating reduced expression in KD and KD+AFD samples and elevated expression in AFD compared to Ctrl. Paired comparisons using Student's *t*-test and FDR correction. ***p-value<0.001.

(E) Venn diagram shows overlap of GSEA-enriched pathways ($p \leq 0.05$), in cancer cells of the scArg-screen, comparing KD vs. Ctrl and AFD vs. Ctrl (left); heatmap shows shared enriched pathways (right). The full list of common enriched pathways is provided in **Table S1**. NES - normalized enriched score.

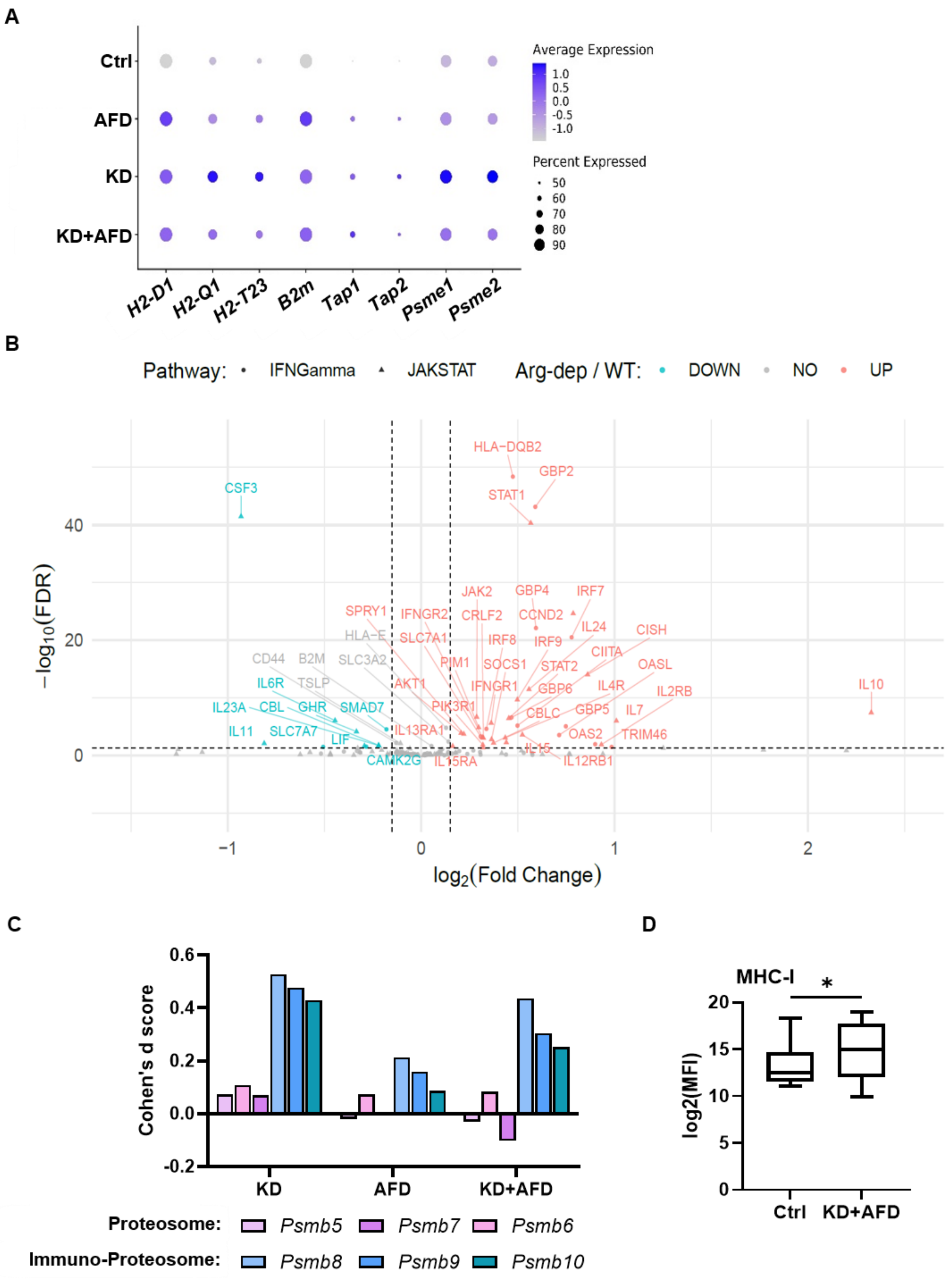
A
Ctrl
AFD
KD
KD+AFD
H2-D1
H2-Q1
H2-T23
B2m
Tap1
Tap2
Psme1
Psme2
Average Expression
1.0
0.5
0.0
-0.5
-1.0
Percent Expressed
50
60
70
80
90
B
Pathway: IFNGamma JAKSTAT
Arg-dep / WT: DOWN NO UP
HLA-DQB2
GBP2
STAT1
CSF3
JAK2
GBP4
IRF7
SPRY1
IFNGR2
CRLF2
CCND2
IL24
CISH
HLA-E
SLC7A1
IRF8
IRF9
CIITA
CD44
B2M
SLC3A2
PIM1
SOCS1
STAT2
OASL
IL6R
TSLP
AKT1
IFNGR1
GBP6
IL4R
IL2RB
IL10
IL23A
CBL
GHR
SMAD7
PIK3R1
CBLC
GBP5
IL7
IL11
SLC7A7
IL13RA1
OAS2
TRIM46
LIF
IL15
CAMK2G
IL15RA
IL12RB1
−log10(FDR)
log2(Fold Change)
40
20
0
−1
0
1
2
C
Cohen's d score
0.6
0.4
0.2
0.0
-0.2
KD
AFD
KD+AFD
Proteosome: Psmb5 Psmb7 Psmb6
Immuno-Proteosome: Psmb8 Psmb9 Psmb10
D
MHC-I
*
log2(MFI)
20
15
10
5
0
Ctrl
KD+AFD

Figure S3. Increased in vivo IFNγ/JAK-STAT signaling

(A) Dot plot based on the scArg-screen shows increased expression of IFNγ-regulated antigen presentation genes in cancer cells following arginine restriction. All the comparisons of treatments to Ctrl are significant for these genes (except for AFD vs. Ctrl for *Psme2*), with adjusted-p-value<0.0001.

(B) Volcano plot based on the scArg-screen demonstrates differentially expressed genes (DEGs) from IFNγ (circles) and JAK–STAT (triangles) pathways in KD+AFD vs. Ctrl cancer cells. Red = upregulated; blue = downregulated. Genes shown pass FDR < 0.05 and |logFC| > 0.15. DEGs were calculated following pseudo-bulking of each treatment single sample into 12 pseudo-samples.

(C) Bar graph based on the scArg-screen shows stronger induction of immunoproteasome (IP) vs. proteasome subunits following arginine deprivation, as measured by Cohen's d effect sizes.

(D) Flow cytometry shows elevated MHC-I expression in tumor cells ($CD326^+$) from KD+AFD vs. Ctrl mice. n = 14–15, 3 experiments. two-way ANOVA. * $p = 0.021$.

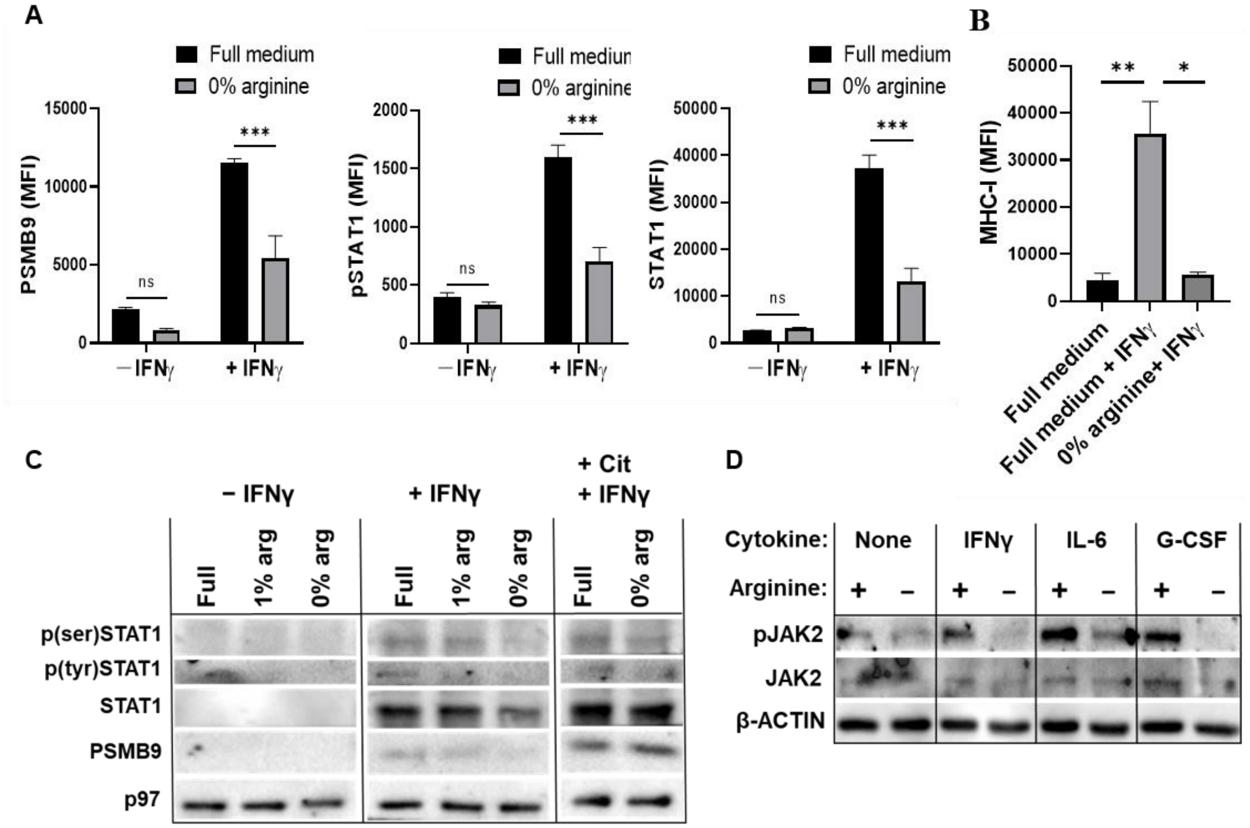

Figure S4. Arginine deprivation suppresses IFNγ signaling in vitro

(A) Flow cytometry demonstrates that 4T1 cells under arginine deprivation fail to induce STAT1 **(left)**, p(Ser)STAT1 **(middle)** and PSMB9 **(right)** in response to IFNγ supplementation. n = *3*. Two-way ANOVA and Tukey tests. p-values <0.001.

(B) Flow cytometry demonstrates that 4T1 cells under arginine deprivation fail to induce MHC-I in response to IFNγ. *n* = 2. One-way ANOVA and Tukey tests. p-values: 0.0095 and 0.011.

(C) Western blot shows reduced expression of STAT1, pSTAT1 (Tyr and Ser), and PSMB9 in 4T1 spheroids under arginine deprivation, which can be rescued by citrulline supplementation.

(D) Western blot demonstrates that pJAK2 is induced by IFNγ, IL-6, and G-CSF only in the presence of arginine, showing dependency on extracellular arginine for pathway activation.

NS - not significant, * $p < 0.05$; ** $p < 0.01$; *** $p < 0.001$.

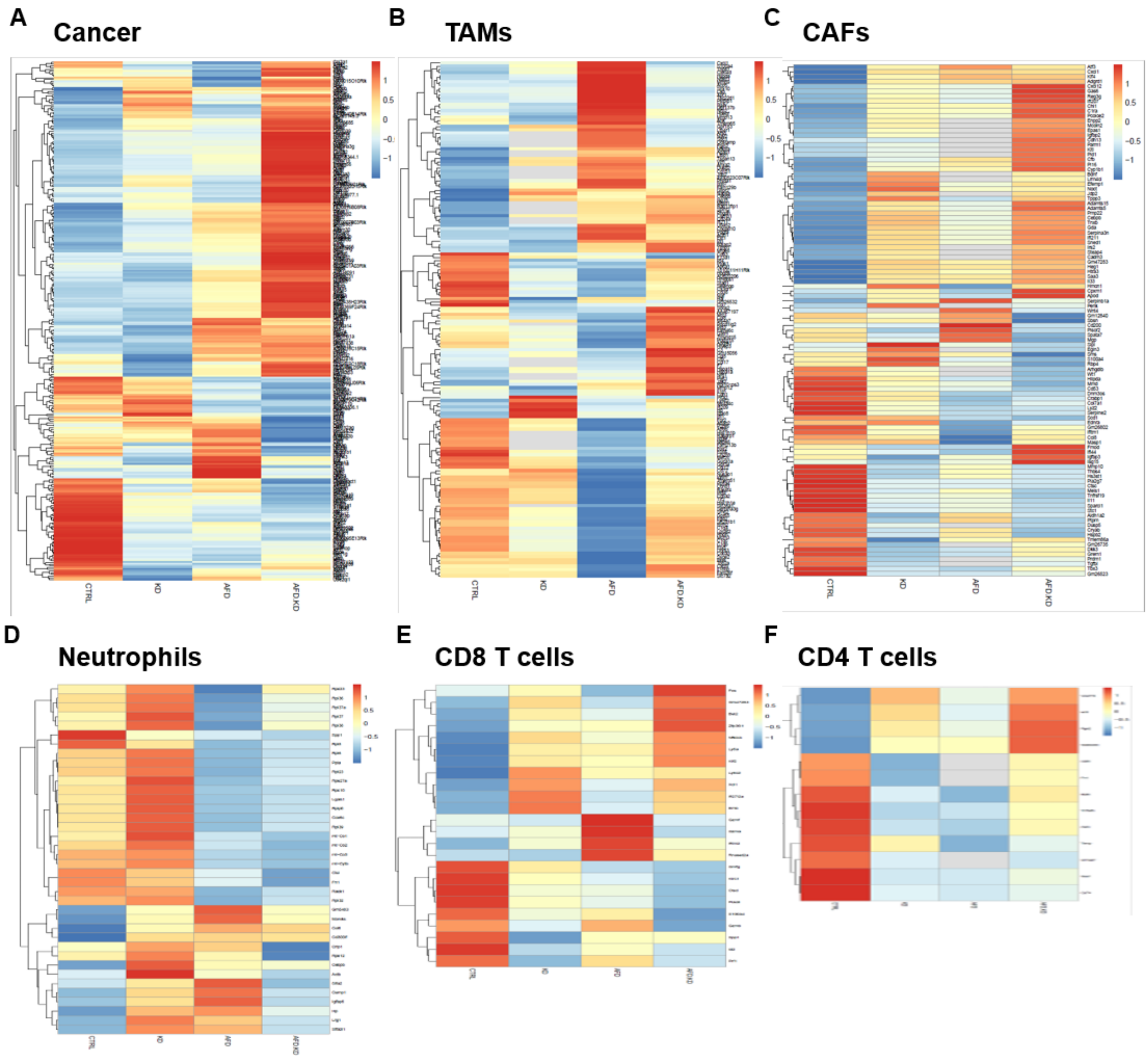

Figure S5. Cell-type-specific transcriptional responses to arginine deprivation

(A-F). Heatmaps showing DEGs with *padj* < *0.05*, baseMean > 5, and |log2FC| > 1 in the TME cell populations identified in the scArg-screen, demonstrating robust transcriptional responses to arginine deprivation: **(A)** Cancer cells (301 genes), **(B)** TAMs (200 genes), **(C)** CAFs (105 genes), **(D)** Neutrophils (38 genes), **(E)** CD8+ T cells (24 genes), **(F)** CD4+ T cells (13 genes). Z-score normalized baseMean expression values are shown across Ctrl, KD, AFD, and KD+AFD groups. DEGs were identified using pseudo-bulking into 12 pseudo-samples per treatment group.

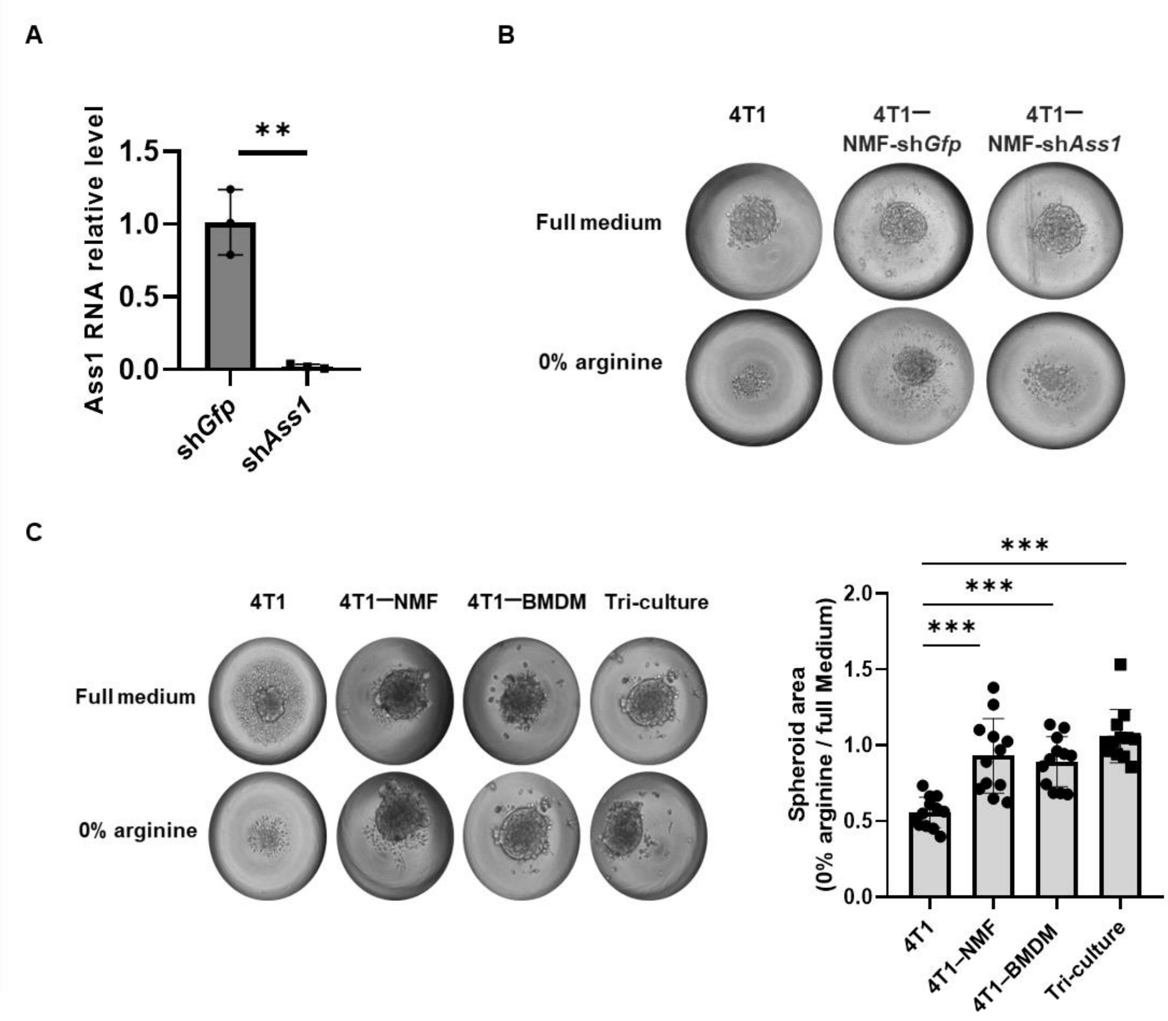

Figure S6. Fibroblast- and macrophage-mediated support preserves cancer cell survival under arginine deprivation

(A) RT-PCR analysis shows reduced *Ass1* expression in NMFs following sh*Ass1* knockdown. n = 3. Student's t-test. p-value: 0.0016.

(B) Representative images of 4T1 monoculture (4T1) or 4T1 co-cultured with NMFs expressing sh*Gfp* (NMF-sh*Gfp*) or sh*Ass1* (NMF-sh*Ass1*). Preserved spheroid growth under arginine-deprived conditions was observed only when 4T1 cells were co-cultured with NMFs expressing ASS1. n = 3. Four spheroids were analyzed per replicate. One-way ANOVA and Tukey tests.

(C) Representative images **(left)** and quantification **(right)** of 4T1 cells co-cultured with NMFs, BMDMs, or both, demonstrating preserved spheroid size under arginine-deprived conditions. n = 3. Four spheroids were analyzed per replicate. One-way ANOVA and Tukey tests.

** $p < 0.01$; *** $p < 0.001$.

Pathway (Hallmark Gene Set)	Average −log10(q) across KD, KD-AFD, AFD vs Ctrl	General Function in Adaptive Cancer Survival	TNBC-Specific Relevance
INTERFERON_GAMMA_RESPONSE	4	Sustained IFNγ signaling selects for antigen-presentation or JAK/STAT-escape variants that survive cytotoxic immune pressure (31).	Drives proliferation, invasion, PD-L1 upregulation, and resistance under immune and therapeutic pressure (31).
INTERFERON_ALPHA_RESPONSE	4	IFN-stimulated genes enhance DNA-damage tolerance, stress adaptation, and anti-apoptotic survival (31).	Marks inflamed tumors but contributes to adaptive resistance and checkpoint upregulation after therapy (31).
ALLOGRAFT_REJECTION	3.49	Strong cytotoxic immune pressure enriches for antigen-loss or checkpoint-high clones that evade clearance (32).	Immune-infiltrated TNBC displays allograft-like signatures that promote immune evasion during chemo-immunotherapy (32).
UNFOLDED_PROTEIN_RESPONSE	1.72	UPR buffers ER stress from hypoxia, nutrient shortage, and therapy via IRE1/XBP1-mediated survival pathways (33).	Supports hypoxic adaptation, stem-like programs, and chemoresistance in aggressive TNBC(33).
MYC_TARGETS_V1	1.57	Enhances anabolic metabolism and redox balance to sustain rapid growth under metabolic/genotoxic stress (34).	MYC-driven metabolism and transcriptional reprogramming promote proliferation and therapy resistance (34).
IL6_JAK_STAT3_SIGNALING	0.99	Activates anti-apoptotic and inflammatory programs and creates an immunosuppressive niche (31,35).	STAT3 enhances stemness, survival under cytotoxic stress, and immune evasion in TNBC (31,35).

Table S6. Consistently enriched Hallmark pathways across KD, KD-AFD and AFD vs. Ctrl comparisons, with roles in cancer cell adaptive survival and TNBC-specific evidence
Pathways shown are those enriched (FDR $q < 0.25$) in all three comparisons (KD vs. Ctrl, KD-AFD vs. Ctrl, AFD vs. Ctrl) in the cancer cells of the scArg screen. Average −log10(q) represents the arithmetic mean enrichment score across the three comparisons. Biological roles highlight known adaptive survival mechanisms enabling cancer cells to persist under immune, metabolic and therapeutic stress. TNBC-specific relevance summarizes mechanisms supporting tumor cell survival, stemness and therapy resistance in TNBC [31–35].

Table S1. (Separate file)
Shared GSEA-enriched pathways ($p \leq 0.05$) in KD vs. Ctrl and AFD vs. Ctrl cancer cells from 4t1 tumors of the scArg-screen. NES - normalized enriched score.

Table S2. (Separate file)

Correlation of *ASS1* expression with IFNγ/JAK–STAT pathway genes and activity. "All" indicates the average expression of all pathway genes, and "activity" reflects pathway activity estimated by ssGSEA.

Table S3. (Separate file)

GSEA-enriched pathways ($p \leq 0.05$) shared between cancer cells and CAFs or cancer cells and TAMs, based on the scArg-screen. NES - normalized enriched score.

Table S4. (Separate file)

Enriched ligand-receptor interactions of high-*ASS1* tumors, based on TCGA breast cancer samples.

Table S5. (Separate file)

Enriched ligand-receptor interactions of low-*ASS1* tumors, based on TCGA breast cancer samples.

Western blot source file

Uncropped western blots corresponding to the main-figure panels. The exposures used in the final figure are marked with red frames. Molecular-weight markers are indicated, and band-intensity values and ratios compared to the housekeeping gene are provided in the accompanying table.

References

31. Hu, X.; li, J.; Fu, M.; Zhao, X.; Wang, W. The JAK/STAT Signaling Pathway: From Bench to Clinic. *Signal Transduction and Targeted Therapy 2021 6:1* 2021, *6*, 402-, doi:10.1038/s41392-021-00791-1.
32. Schreiber, R.D.; Old, L.J.; Smyth, M.J. Cancer Immunoediting: Integrating Immunity's Roles in Cancer Suppression and Promotion. *Science (1979)* 2011, *331*, 1565–1570, doi:10.1126/SCIENCE.1203486;SUBPAGE:STRING:ABSTRACT;ISSUE:ISSUE:DOI.
33. Hetz, C.; Papa, F.R. The Unfolded Protein Response and Cell Fate Control. *Mol Cell* 2018, *69*, 169–181, doi:10.1016/J.MOLCEL.2017.06.017.
34. Stine, Z.E.; Walton, Z.E.; Altman, B.J.; Hsieh, A.L.; Dang, C. V. MYC, Metabolism, and Cancer. *Cancer Discov* 2015, *5*, 1024–1039, doi:10.1158/2159-8290.CD-15-0507/43152/P/MYC-METABOLISM-AND-CANCERMYC-METABOLISM-AND-CANCER.
35. Johnson, D.E.; O'Keefe, R.A.; Grandis, J.R. Targeting the IL-6/JAK/STAT3 Signalling Axis in Cancer. *Nat Rev Clin Oncol* 2018, *15*, 234–248, doi:10.1038/NRCLINONC.2018.8;SUBJMETA.
