# Supplementary material for: Inhibition of Tumor Microenvironment-Driven JAK-STAT Signaling Enhances Response to Arginine Deprivation Therapy in Triple-Negative Breast Cancer
Source: Cells. 2025 Dec 23;15(1):25. doi: 10.3390/cells15010025 (PMC12785028; doi:10.3390/cells15010025)
Supplement: Supplementary file 1 [file cells-15-00025-s001.zip › Supp cells-4016445 proof/Cells-4016445 Supplementary Methods.pdf]

Supplementary Methods

A full description of all experimental procedures and detailed protocols is provided below; the conceptual framework and overall experimental design are summarized in the Main Methods section.

1. *In vivo Animal Studies*

Animal experiments were approved by the Weizmann Institute Animal Care and Use Committee, following the guidelines of the U.S. National Institutes of Health, the European Commission, and Israeli regulations (IACUC numbers 00810122-3 and 02000324-3). Mice were randomly assigned to experimental groups, investigators were not involved in allocation, and all procedures followed predefined humane endpoints. Animals were monitored daily for distress, weight loss, and tumor burden. Sample sizes were determined based on prior publications and internal pilot data, and no unexpected adverse events occurred. All inclusion/exclusion criteria, humane endpoints, and monitoring procedures were established before experiments began. BALB/c mice were purchased from Envigo Laboratories (Indianapolis, IN, USA) and maintained under specific-pathogen-free conditions at the Weizmann Institute of Science animal facility. To generate a syngeneic orthotopic mouse breast cancer model, 8–10-week-old female BALB/c mice were randomly assigned to experimental groups. Mice were injected with 0.3×10^6 4T1, 4T1-shGfp, or 4T1-shAss1 cells (in PBS) into the lower right mammary fat pad. Between days 15 and 21 post-tumor inoculation, mice were sacrificed; tumors were resected and weighed. Tumors were then subjected to further analyses, including flow cytometry, western blotting, and single-cell RNA sequencing. In some experiments, terminal bleeding was performed prior to tumor collection to analyze amino acid levels by LC–MS. An arginine-free diet (AFD) was administered to mice 7–9 days before tumor inoculation, and for specific experiments, AFD was introduced on day 11 post-inoculation. For experiments involving ruxolitinib treatment, BALB/c female mice were injected with 4T1-shGfp or 4T1-shAss1 cells. On day 11, mice were grouped by similar tumor sizes and subjected to treatment with either an arginine-free or regular diet, combined with oral gavage of ruxolitinib (40 mg/kg) three times per week. The gavage solution was prepared by filtering a mix containing 6% ruxolitinib dissolved in DMSO, 40% PEG300 (MedChemExpress, Monmouth Junction, NJ, USA), 5% Tween 80 (Sigma-Aldrich, St. Louis, O, USA), and 49% 0.9% NaCl saline.

2. *Flow Cytometry of 4T1 Tumors*

Excised 4T1 tumors were minced using gentleMACS C-tubes (Miltenyi Biotec, Bergisch Gladbach, Germany) and enzymatically digested in RPMI containing 3 mg/mL collagenase A (Sigma-Aldrich, St. Louis, MO, USA) and 1 mg/mL (70 U/mL) DNase I (Sigma-Aldrich, St. Louis, MO, USA) for 40 minutes at 37 °C. Following digestion, tumors were further minced with gentleMACS, filtered through a 70 µm strainer, and centrifuged at 4 °C. Cell pellets were treated with red blood cell lysis buffer (BioLegend, San Diego, CA, USA) for 5 minutes at room temperature, neutralized with PBS containing 0.5% BSA and 2 mM EDTA (FACS buffer), counted, and centrifuged. Negative controls, single-stained controls, and FMO controls were included to ensure robust gating and compensation across experiments. Gating strategies were pre-defined and kept constant across biological repeats to ensure reproducibility. Cells were incubated with Fc blocker (BioLegend, San Diego, CA, USA) at 1:100 for 10 minutes, then stained for surface antigens on ice for 20 minutes. Surface antibodies included anti-CD326–APC/Cy7 and anti-MHC I–FITC for tumor cells, and anti-CD45–PE/Cy7, anti-CD11b–BV605, and anti-F4/80–PE for macrophages (all BioLegend, San Diego, CA, USA). Samples were washed twice with FACS buffer, filtered, and stained with DAPI before acquisition. Data were collected on a CytoFLEX cytometer (Beckman Coulter, Brea, CA, USA) and analyzed with FlowJo software (FlowJo LLC, Ashland, OR, USA).

3. *NMF Production*

Normal mammary fat pad fibroblasts (NMFs) were isolated from the mammary fat pads of two 12-week-old female BALB/c mice per biological replicate. The fat pads were minced and dissociated using gentleMACS C-tubes (Miltenyi Biotec, Bergisch Gladbach, Germany) in RPMI containing 1 mg/mL collagenase II and 1 mg/mL collagenase IV (Worthington Biochemical Corporation, Lakewood, NJ, USA) and 0.1 mg/mL DNase I (Sigma-Aldrich, St. Louis, MO, USA). Samples were incubated for 40 minutes at 37 °C with subsequent mechanical dissociation. The reaction was stopped by adding DMEM (Gibco, Thermo Fisher Scientific, Waltham, MA, USA) supplemented with 10% FBS (Sigma-Aldrich, St. Louis, MO, USA). Cells were filtered through a 70 μm strainer and pelleted by centrifugation at 4 °C. Pellets were treated with RBC lysis buffer (Sigma-Aldrich, St. Louis, MO, USA) for 5 minutes at room temperature, washed with PBS, and centrifuged. Fibroblasts were seeded on 10 cm plates pre-coated with 4.25 mg/mL rat-tail type I collagen (Sigma-Aldrich, St. Louis, MO, USA) and expanded for 6 days in RPMI supplemented with 10% FBS, 1% glutamine, and 1% penicillin/streptomycin (Sartorius, Göttingen, Germany), with media replenished every 3 days.

4. *BMDM Production*

Bone marrow-derived macrophages (BMDMs) were isolated by flushing femurs and tibias of adult mice with PBS using a 27G needle. Cells were filtered through a 100 μm strainer, centrifuged (300 × g, 5 min, 4 °C), and treated with 1 mL red blood cell lysis buffer (BioLegend, San Diego, CA, USA; diluted 1:10 in ddH_2O) for 5 minutes on ice. After washing with 30 mL PBS, cells were plated in non–tissue-culture-treated 10 cm petri dishes in full DMEM medium supplemented with 10% FBS and 1% Pen/Strep, and 20% L929-conditioned medium. After 3 days, an additional 5 mL of medium was added. On day 6, cells were transferred to tissue-culture-treated plates, and experiments were performed on day 7. L929-conditioned medium was prepared from L929 fibroblasts (ATCC, Manassas, VA, USA) and stored at −20 °C until use.

5. *Cell Lines*

4T1 cells (CRL-2539™, ATCC, Manassas, VA, USA) were kindly provided by Prof. Moshe Oren (Weizmann Institute of Science) and Prof. Ruth Scherz-Shouval (Weizmann Institute of Science). GFP-expressing 4T1 cells, generated via the FUW-GFP vector, were kindly provided by Prof. Ruth Scherz-Shouval (Weizmann Institute of Science). Cells were cultured in RPMI medium (Invitrogen, Thermo Fisher Scientific, Waltham, MA, USA) supplemented with 10% heat-inactivated fetal bovine serum (FBS; Sigma-Aldrich, St. Louis, MO, USA), 1% penicillin–streptomycin, and 2 mM glutamine (RPMI full medium). RAW264.7 cells (TIB-71, ATCC, Manassas, VA, USA) were cultured in DMEM (Gibco, Thermo Fisher Scientific, Waltham, MA, USA) supplemented with 10% heat-inactivated FBS, 1% penicillin–streptomycin, and 2 mM glutamine (DMEM full medium). All cells were cultured at 37 °C in a humidified incubator with 5% CO_2, maintained below passage 10, and routinely tested for Mycoplasma using an EZ-PCR test kit (Biological Industries, Beit HaEmek, Israel).

6. *Virus Infection*

Cancer cells and NMFs were infected with pLKO-based lentiviral vectors carrying shRNA sequences targeting Gfp (non-target control) or mouse Ass1 (Dharmacon, Horizon Discovery, Cambridge, UK). Infected cells were selected with 2 μg/mL puromycin, and ASS1 downregulation was validated by RT–PCR and rescue survival experiments with citrulline.

7. *Arginine-Depleted Plasmax Medium*

Arginine- and citrulline-free Plasmax medium was prepared as previously described[16]. To minimize contributions from serum components, all experiments used 2.5% dialyzed FBS (IMBH, Beit HaEmek, Israel). For full Plasmax medium, 1.14 mM L-arginine (Sigma-Aldrich, St. Louis, MO, USA) was added. For rescue experiments, 2 mM citrulline (Sigma-Aldrich, St. Louis, MO, USA) was supplemented. Cytokines IFNγ (20 ng/mL), IL-6 (10 ng/mL), and G-CSF (25 ng/mL) (all PeproTech, Rehovot, Israel) were added as indicated. For In vitro experiments with ruxolitinib (MedKoo Biosciences, Morrisville, NC, USA), 24 μM was added 24 hours before analysis, dissolved in DMSO.

8. *3D Culturing of 4T1 Cells*

For 3D culturing, 0.554 × 10^6 or 1.731 × 10^6 4T1 cells were seeded in Elplasia 24-well or 6-well plates (Corning, Corning, NY, USA), respectively (600 cells per microwell). Cells were cultured in arginine/citrulline-free Plasmax medium supplemented with arginine and cytokines according to each experimental design (see figure legends).

9. *3D Co-Culture and Tri-Culture with Primary Cells*

For 3D co-culture of 4T1 cells with normal mammary fibroblasts (NMFs), 0.139 × 10^6 4T1 cells (250 cells/microwell) and 0.416 × 10^6 NMFs (750 cells/microwell) were seeded into Elplasia 24-well plates (Corning, Corning, NY, USA). Single cultures of 0.332 × 10^6 4T1 cells (600 cells/microwell) or 0.416 × 10^6 NMFs (750 cells/microwell) were seeded separately. For 4T1–BMDM co-cultures, 0.332 × 10^6 cells of each type (600 cells/microwell) were seeded. In tri-culture experiments, 0.139 × 10^6 4T1 cells, 0.139 × 10^6 BMDMs (each 250 cells/microwell), and 0.332 × 10^6 NMFs (600 cells/microwell) were co-seeded. Cells were cultured in 1 mL arginine/citrulline-free Plasmax medium supplemented with arginine or cytokines for 48 hours. For rescue experiments, NMF-conditioned medium was collected after 48 hours, filtered through a 0.22 μm syringe filter (Millipore, Burlington, MA, USA), and used to culture 4T1 spheroids for 48 hours. Seeding ratios and culture times were selected based on pilot experiments demonstrating robust and reproducible spheroid formation.

10. *Co-Culture of 4T1 Cells and RAW264.7 Macrophages*

RAW264.7 macrophages were seeded into 25 cm^2 flasks in complete DMEM medium. On the same day, 1.0 × 10^6 or 1.5 × 10^6 4T1 cells were seeded into 12-well plates in complete RPMI medium for future culture in either full medium or arginine/citrulline-free Plasmax medium, respectively. The following day, 0.55 × 10^6 RAW264.7 macrophages were reseeded into cancer cell wells in arginine/citrulline-free Plasmax medium. Selected wells were supplemented with arginine and/or IFNγ according to the experimental design. Cells were harvested for flow cytometry 48 hours after macrophage addition.

11. *Flow Cytometry of In Vitro Samples*

Flow cytometry was performed on a CytoFLEX system (Beckman Coulter, Brea, CA, USA) and analyzed using FlowJo software (FlowJo LLC, Ashland, OR, USA). Negative controls, single-stained controls, and fluorescence minus one (FMO) controls were included in each experiment to compensate for spectral overlap and identify cell populations. The gating strategy was kept constant across experimental repeats.

11.1 Spheroid Preparation

4T1, NMF, or 4T1–NMF spheroids were collected by repeated pipetting from Elplasia plates, transferred to Eppendorf tubes, and washed twice with cold PBS. Spheroids were resuspended in 200 μL Accumax solution (Sigma-Aldrich, St. Louis, MO, USA), transferred to 96-well round-bottom plates, and

incubated at 37 °C for 30–40 minutes with pipetting every 5 minutes to aid dissociation. After dissociation, cells were centrifuged and washed once with FACS buffer.

11.2 2D Culture Preparation

4T1 and RAW264.7 cells were washed with PBS and incubated with TrypLE (Reagent Partners, Rhenium, Israel) at 37 °C for 5 minutes. After incubation, FACS buffer was added, and cells were dissociated by pipetting, transferred to 96-well plates, centrifuged, and washed once with FACS buffer.

11.3 Apoptosis Analysis

Single-cell suspensions were incubated on ice for 20 minutes with antibodies against CD326–APC/Cy7 (BioLegend, San Diego, CA, USA) and PDPN–PerCP/Cy5.5 (BioLegend, San Diego, CA, USA) to detect cancer cells and NMFs, respectively. Cells were washed with FACS buffer and resuspended in 1 μL Annexin V–APC (BioLegend, San Diego, CA, USA) and 49 μL binding buffer (BioLegend, San Diego, CA, USA) for 10 minutes at room temperature. Subsequently, 150 μL binding buffer containing 2 μL DAPI (1:100 dilution) was added.

11.4 Surface and Cytoplasmic Antigen Staining

Cells were stained for surface antigens on ice for 20 minutes, washed twice with FACS buffer, and fixed in 1.6% paraformaldehyde at room temperature for 10 minutes. After washing, cells were permeabilized by dropwise addition of 200 μL TruePhos buffer (BioLegend, San Diego, CA, USA) with gentle vortexing and stored at −20 °C until intracellular staining. For intracellular staining, cells were washed twice and incubated for 30 minutes at room temperature with primary or conjugated antibodies, washed, incubated with secondary antibodies for 20 minutes, washed again, and resuspended in 200 μL FACS buffer. Surface antibodies included anti-MHC I–FITC for cancer spheroids, and anti-CD45–PE/Cy7 and anti-CD326–APC/Cy7 for RAW264.7–cancer co-cultures. Cytoplasmic antibodies included anti-STAT1–PE (1:200, Cell Signaling Technology, Danvers, MA, USA), anti-pSTAT1–PE/Cy7 (1:700, BioLegend, San Diego, CA, USA), and anti-PSMB9 (1:500, Abcam, Cambridge, UK). The secondary antibody was anti-rabbit AF647 (1:2000, BioLegend, San Diego, CA, USA). For co-cultures of cancer cells and NMFs, 4T1–GFP cells were used to distinguish cancer cells.

12. LC–MS Analysis of Amino Acid Levels

Tumor tissue was extracted with 500 μL of 4% aqueous perchloric acid using a pestle motor mixer (Argos Technologies, Elgin, IL, USA), followed by shaking (2000 rpm, 4 °C, 30 minutes). After centrifugation (10,000 g, 10 minutes), a 10 μL aliquot of the supernatant was diluted with 70 μL of 0.1% formic acid. Internal standards [$^{13}C_6$]-arginine (10 μL, 60 μM) and norleucine (10 μL, 10 μM) were added, and a 10 μL aliquot was derivatized with AQC reagent.

Plasma (20 μL) was extracted with 140 μL methanol containing internal standards [$^{13}C_6$]-arginine (10 μL, 600 μM) and norleucine (5 μL, 200 μM) and incubated at 10 °C for 20 minutes. After centrifugation (14,000 g, 5 minutes), the supernatant was concentrated in a SpeedVac concentrator by methanol evaporation, and the residue was resuspended in 0.1% formic acid to a final volume of 100 μL. A 10 μL aliquot was derivatized with AQC reagent.

Cell pellets were extracted with acetonitrile/methanol/water (200:200:100 μL). The suspension was vortexed, incubated at 4 °C for 10 minutes, and centrifuged (21,000 g, 5 minutes). The supernatant was collected, and a 10 μL aliquot was mixed with 10 μL of a labeled amino acid mixture (Sigma-Aldrich, product no. 767964; diluted 1:10,000 in 50% aqueous acetonitrile) as internal standards and derivatized with AQC reagent. Samples were filtered through 0.2 μm PES nanoFilter vials (Thomson Instrument Company, Oceanside, CA, USA) before analysis.

Medium samples (10 μL) were mixed with 10 μL of the labeled amino acid mixture (Sigma-Aldrich, product no. 767964; diluted 1:10,000 in 50% aqueous acetonitrile) as internal standards, derivatized with AQC reagent, and filtered through 0.2 μm PES nanoFilter vials (Thomson Instrument Company, Oceanside, CA, USA) before analysis.

The L-amino acid standard mixture (Sigma-Aldrich, product no. A9906) at concentrations ranging from 0.01 to 10 μM was derivatized with AQC reagent and used to generate standard curves.

Survival Assay by XTT

Twenty thousand 4T1-shGfp and 4T1-shAss1 cells were seeded in 100 μL complete RPMI medium in 96-well flat-bottom plates. The next day, cells were washed with PBS, and the medium was replaced with arginine/citrulline-free Plasmax medium. On the following day, arginine (1.14 mM) and citrulline (2 mM) were added. Cell survival was measured using an XTT kit (Biological Industries, Beit HaEmek, Israel) according to the manufacturer's instructions at 18 hours (baseline) and 4 days post-seeding.

13. *Western Blotting*

Tumor pieces and cell cultures were lysed in RIPA buffer (Sigma-Aldrich, St. Louis, MO, USA) supplemented with 1% protease inhibitor cocktail (Calbiochem, San Diego, CA, USA) and 1% phosphatase inhibitor cocktail (product no. P5726, Sigma-Aldrich, St. Louis, MO, USA). Lysates were centrifuged at 14,000 rpm for 20 minutes at 4 °C, and protein concentration was measured using a BCA assay (Thermo Fisher Scientific, Waltham, MA, USA). Samples (10–20 μg) were resolved on 10% SDS–PAGE gels and transferred to cellulose nitrate membranes (Tamar, Jerusalem, Israel). Membranes were blocked for 1 hour at room temperature with TBST containing 5% skim milk or 5% BSA and then incubated with primary antibodies against ASS1 (1:1000, ab124465, Abcam, Cambridge, UK), β-actin (1:1000, A5441, Sigma-Aldrich, St. Louis, MO, USA), pJAK2 (1:500, 37315, Cell Signaling Technology, Danvers, MA, USA), JAK2 (1:1000, 3230S, Cell Signaling Technology, Danvers, MA, USA), p-STAT1 (Tyr) (1:1000, 9167S, Cell Signaling Technology, Danvers, MA, USA), p-STAT1 (Ser) (1:200, BL686401, BioLegend, San Diego, CA, USA), STAT1 (1:1000, sc-346, Santa Cruz Biotechnology, Dallas, TX, USA), PSMB10 (1:1000, ab183506, Abcam, Cambridge, UK), and PSMB9 (1:2000, ab242061, Abcam, Cambridge, UK). Detection was performed using peroxidase-conjugated goat anti-rabbit or goat anti-mouse IgG secondary antibodies (Jackson ImmunoResearch, West Grove, PA, USA) and enhanced chemiluminescence reagents (ECL HRP substrate, LifeGene, Ness Ziona, Israel). Gels were imaged using a Gel Doc XR+ system (Bio-Rad, Hercules, CA, USA) and analyzed with ImageLab software (version 5.1, Bio-Rad). Band intensities were normalized to loading controls.

14. *RNA Processing and Quantitative PCR (qPCR)*

RNA was extracted from 4T1 3D spheroids using the Direct-zol™ MicroPrep Plus Kit (Zymo Research, Irvine, CA, USA) and from 2D cultures using the RNeasy Mini Kit (Qiagen, Hilden, Germany). Complementary DNA was synthesized from 1 μg RNA using the qScript cDNA Synthesis Kit (Quantabio, Beverly, MA, USA). Quantitative PCR was performed using SYBR Green PCR Master Mix (Thermo Fisher Scientific, Waltham, MA, USA) with the following primers: m*Cat1* (SLC7A1) forward, CTCCTGGCTTACTCTTTGGTGG, reverse, GATCTAGCTCCTCGGTGGTTCT; m*Ass1* forward, TGTTCGCCACTGTATCCAGAAGT, reverse, CCGCTCCTCTTTGTCAGGGTCTA; m*Asl* forward, CGGACTCCTGATGACCCTCAAGT, reverse, TGTACTGTTCCACGCTGTG. All qPCR primer efficiencies were validated prior to use, and melt curves were inspected for specificity.

15. *Quantification of Spheroid Area*

Microscopy images were acquired using an EVOS M5000 microscope (Thermo Fisher Scientific, Waltham, MA, USA) equipped with a 10× fluorite long working distance objective (numerical aperture 0.30, working distance 7.13 mm). Spheroid areas were quantified by manually annotating spheroid boundaries using QuPath software (version 0.5.1) or ImageJ (version 1.54g), followed by area calculation based on pixel measurements. For each replicate, the spheroid area in 0% arginine medium was normalized to the corresponding spheroid area in full medium, and the resulting ratio was used for analysis.

16. *Single-Cell RNA-seq Library Preparation of the scArg-Screen*

Single-cell suspensions of 4T1 tumors were prepared as described above for flow cytometry of primary tumors, followed by red blood cell removal using RBC lysis buffer (BioLegend, San Diego, CA, USA). Cells were counted, and one pool per treatment was generated by combining equal numbers of cells from each tumor (five mice per treatment). Dead cells were removed using a Dead Cell Removal Kit (Miltenyi Biotec, Bergisch Gladbach, Germany), followed by validation with an automated fluorescent cell counter to confirm viability above 70%. Viability thresholds (>70%) and equal cell pooling ensured balanced representation of samples. Single-cell RNA-seq libraries were prepared according to the manufacturer's instructions using the Chromium single-cell RNA-seq platform (10x Genomics, Pleasanton, CA, USA). Briefly, single-cell suspensions were loaded onto a Chromium Controller to generate gel beads in emulsion (GEMs). Within each GEM, cells were lysed, and mRNA was reverse-transcribed into barcoded cDNA. After breaking the emulsion, cDNA was amplified, and libraries were constructed using the Chromium Single Cell 3′ Library Kit (10x Genomics). Final libraries were quantified using the NEBNext Library Quant Kit for Illumina (New England Biolabs, Ipswich, MA, USA) and a high-sensitivity D1000 TapeStation (Agilent Technologies, Santa Clara, CA, USA). Libraries were pooled and sequenced on an Illumina NovaSeq 6000 system (Illumina, San Diego, CA, USA) using an SP 100-cycle reagent kit, targeting approximately 50,000 reads per cell.

17. *Analysis of the Single-Cell RNA-seq Screen*

17.1 Pre-processing and Quality Control

Data were pre-processed using the 10x Genomics Cell Ranger software (v3.1.0; 10x Genomics, Pleasanton, CA, USA) and annotated against the mm10 reference genome (refdata-gex-mm10-2020-A provided by 10x Genomics). Quality control, clustering, and downstream analyses were performed in R (v4.0.0; R Foundation for Statistical Computing, Vienna, Austria) using the Seurat package (v3.2.0). Doublets and multiplets were excluded based on gene and unique molecular identifier (UMI) counts. Putative dead cells and empty droplets were filtered based on mitochondrial gene percentage, gene count, and UMI count. This resulted in a final dataset of 21,873 cells with 18,264 detected genes. Quality control cutoffs (UMI count, gene count, mitochondrial percentage) were pre-defined and applied uniformly across all samples to avoid data-driven thresholding.

17.2 Clustering

Normalization and scaling were performed separately for each sample using the SCTransform function in Seurat. Sample-wise SCTransform normalization followed by integration using SelectIntegrationFeatures effectively minimized batch effects across treatment groups. The top 2,000 highly variable genes were identified and integrated into a single object using the Seurat merge function. Cells were clustered using the FindNeighbors and FindClusters functions with 17 principal components and a resolution parameter of 1.2. Dimensionality reduction and visualization were performed using Uniform Manifold Approximation and Projection (UMAP) implemented via the

RunUMAP function. Cell cluster identities were assigned based on canonical marker gene expression using the FindAllMarkers or FindMarkers functions with default parameters.

17.3 Differential Gene Expression Analysis

Differential gene expression analysis was conducted using the Seurat package and the Wilcoxon rank-sum test to compare experimental conditions. Genes were considered differentially expressed based on a $\log_2$ fold change ≥ 0.25 and a Benjamini–Hochberg false discovery rate (FDR)-adjusted p-value ≤ 0.05 and were visualized using volcano plots generated with the EnhancedVolcano R package (Blighe, K., 2018). For heatmap visualization, scaled (z-score) expression values generated using the SCTransform workflow were plotted using the Seurat DoHeatmap function. Average gene expression per cluster was calculated using the Seurat AverageExpression function on log-normalized scaled data.

17.4 GSEA Pathway Enrichment and Hallmark Analysis

KEGG and Reactome gene set enrichment analyses (GSEA) were performed for unbiased pathway discovery, while Hallmark GSEA was used for focused interpretation of key transcriptional programs enriched in cancer cells. The Gene Set Enrichment Analysis software (v4.2.3; Broad Institute, Cambridge, MA, USA) was used. For Hallmark analysis, differentially expressed genes were ranked by $\log_2$ fold change from highest to lowest, and pre-ranked gene lists were analyzed using 1,000 permutations with the ranked-list mode and appropriate chip platform settings. Analyses were performed against the HALLMARK gene set collection.

17.5 Differential Gene Expression Using Pseudo-Bulking

Pseudo-bulking was performed to avoid inflation of p-values arising from treating individual cells as independent samples. For each comparison and cell type, pseudo-bulk samples were generated by randomly aggregating expression profiles from multiple cells into a single gene expression vector. Differential gene expression was calculated using the DESeq2 R package (v1.44). P-values were adjusted using FDR correction. Pseudo-bulking was applied only to cell types with more than 250 cells, including cancer cells, CAFs, TAMs, CD4 T cells, CD8 T cells, neutrophils, and NK cells. Robustness of the pseudo-bulking approach and the selection of 12 pseudo-bulk samples was assessed using Spearman's correlation of $\log_2$ fold changes and adjusted p-values derived from analyses using varying pseudo-bulk sizes. NK cells were excluded from downstream analyses due to poor correlation.

17.6 Plotting IFNγ–JAK–STAT Differentially Expressed Genes

Single cancer cell expression data were pseudo-bulked to represent control and KD+AFD conditions. Mouse genes were converted to their human homologs using the biomaRt R package (v2.60.1). Differentially expressed genes between conditions were identified, and visualization was performed using the ggplot2 R package (v3.5.1). Genes with FDR < 0.05 were labeled, and genes with $\log_2$ fold change > 0.15 were colored red or blue to indicate upregulation or downregulation, respectively.

18. TCGA–TNBC Correlation Analysis Between ASS1 and IFNγ/JAK–STAT Genes

The triple-negative breast cancer subset of TCGA (TCGA–TNBC) comprised 122 bulk RNA-seq samples. Spearman's correlation coefficients were calculated between ASS1 expression and genes belonging to the IFNγ and JAK–STAT pathways using TPM-normalized expression values. Pathway activity was estimated either by mean expression of pathway genes or by single-sample gene set enrichment analysis (ssGSEA). Correlation p-values were adjusted using FDR correction, and genes with FDR < 0.05 were ranked by correlation coefficient (ϱ).

19. TCGA–BRCA Analysis of ASS1-Low and ASS1-High Tumors

19.1 Cell Fraction Analysis

Deconvolved cell fractions for 11 immune and stromal cell types were obtained from TCGA breast cancer samples as reported by Wang et al. Patient samples were stratified into ASS1-low and ASS1-high groups based on a pre-specified median expression cutoff. Wilcoxon rank-sum tests were applied to compare cell fractions between groups.

19.2 Receptor–Ligand Analysis

Cell type–specific deconvolved gene expression data were obtained from Wang et al. Samples were stratified by ASS1 expression as described above. Ligand–receptor interactions differentially regulated between ASS1-low and ASS1-high tumors were identified using LIRICS. Functional categorization and enrichment of interactions were assessed using Fisher's exact test.

20. Statistical Analysis

Unless otherwise specified, statistical analyses were performed using one-way or two-way ANOVA with Tukey's multiple comparisons test, Student's t-tests (two-tailed), or Wilcoxon rank-sum tests, with Dunnett's correction applied when appropriate. Sample sizes were pre-determined based on standard experimental practices and are specified for each experiment. Experiments included biological and technical replicates and were repeated at least three times unless stated otherwise. Data are presented as mean ± standard deviation. Statistical significance was defined as $p < 0.05$ (*$p < 0.05$, **$p < 0.005$, ***$p < 0.0005$, ****$p < 0.0001$). All statistical analyses used pre-defined thresholds, and assumptions were verified where applicable.
