# Supplementary material for: Inhibition of Tumor Microenvironment-Driven JAK-STAT Signaling Enhances Response to Arginine Deprivation Therapy in Triple-Negative Breast Cancer
Source: Cells. 2025 Dec 23;15(1):25. doi: 10.3390/cells15010025 (PMC12785028; doi:10.3390/cells15010025)
Supplement: Supplementary file 1 [file cells-15-00025-s001.zip › Supp cells-4016445 proof/Cells-4016445 Western blots.pdf]

Figure 2D – original blots

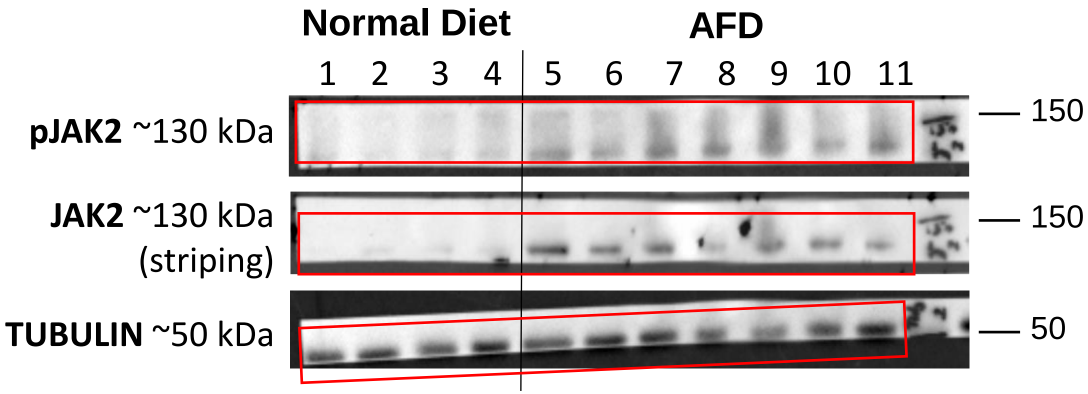

Adj. Volume (Int)

Lane	TUBULIN	pJAK2	Jak2
1	5069875	1779273	277684
2	5377560	1156785	502536
3	5130504	1481450	698208
4	5606940	1640616	581042
5	5909544	4049280	5059440
6	5527500	2957300	2956680
7	5304214	3982600	3591610
8	3966212	3546510	1518682
9	4035051	3724680	2461470
10	4894272	2835816	2701820
11	6016938	3917606	1794430

Ratio (gene/TUBULIN)

Lane	pJAK2	Jak2
1	0.35095	0.05477
2	0.21511	0.09345
3	0.28875	0.13609
4	0.2926	0.10363
5	0.68521	0.85615
6	0.53502	0.5349
7	0.75084	0.67712
8	0.89418	0.3829
9	0.92308	0.61002
10	0.57942	0.55204
11	0.6511	0.29823

Figure 2F – original blots

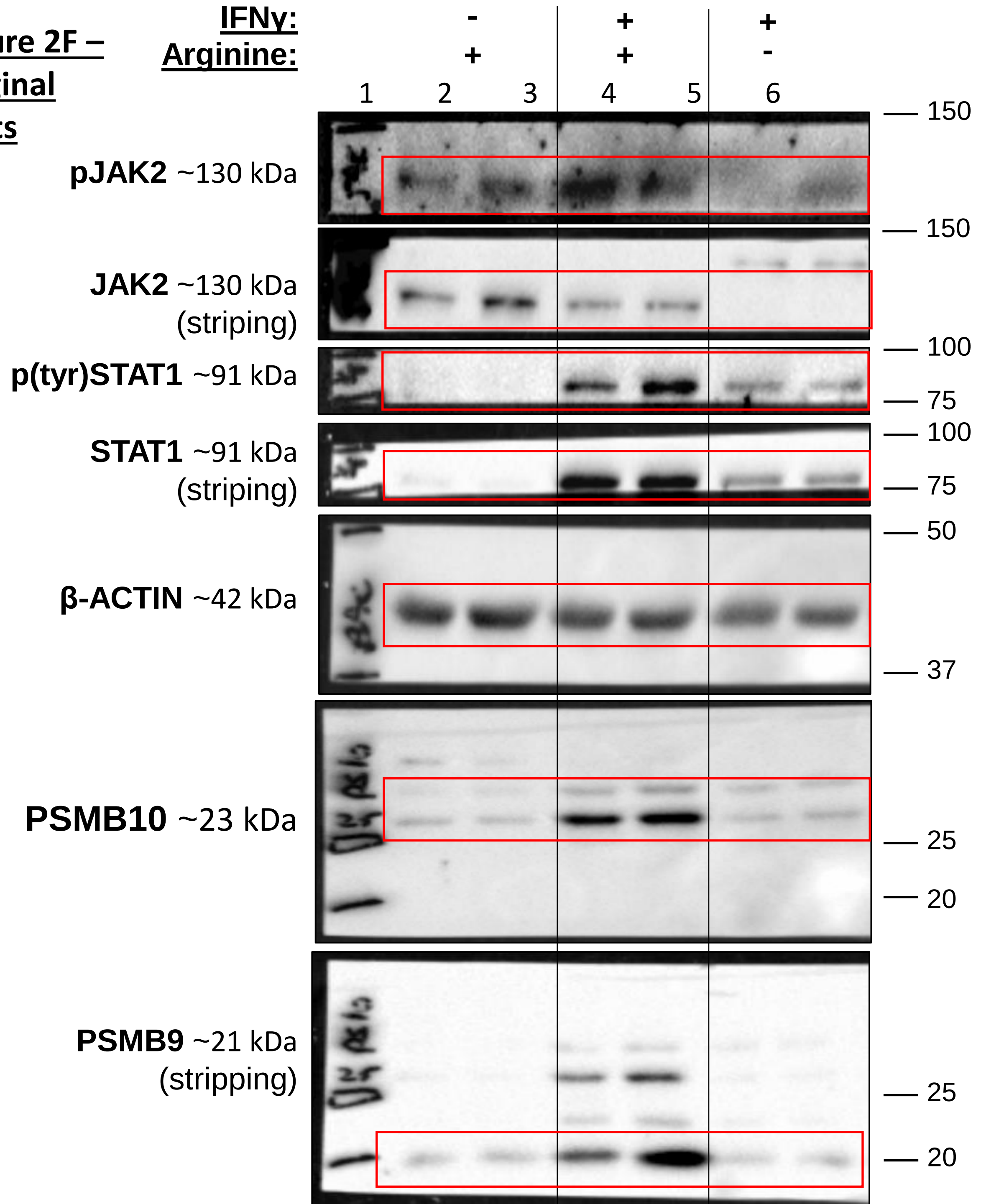

Adj. Volume (Int)

Lane	βactin	pJAK2	JAK2	pSTAT1tyr	STAT1	ps10	ps9
1	5522823	2602326	1795265	72325	44550	900704	816504
2	6314359	2796300	2774979	40925	661368	657384	976690
3	5007120	4795614	1519975	2615166	4754511	4948290	3373748
4	5062889	5243526	1659393	4807264	4710692	6218093	7672050
5	4236470	2776564	18434	1488340	2238444	715285	995760
6	5151611	4028220	75990	987810	2452324	1702126	813726

Ratio (gene/β-ACTIN)

Lane	pJAK2	JAK2	pSTAT1tyr	STAT1	ps10	ps9
1	0.47119	0.32506	0.0131	0.00807	0.16309	0.14784
2	0.44285	0.43947	0.00648	0.10474	0.10411	0.15468
3	0.95776	0.30356	0.52229	0.94955	0.98825	0.67379
4	1.03568	0.32776	0.94951	0.93044	1.22817	1.51535
5	0.6554	0.00435	0.35132	0.52837	0.16884	0.23504
6	0.78193	0.01475	0.19175	0.47603	0.33041	0.15796

Figure S4C – original blots

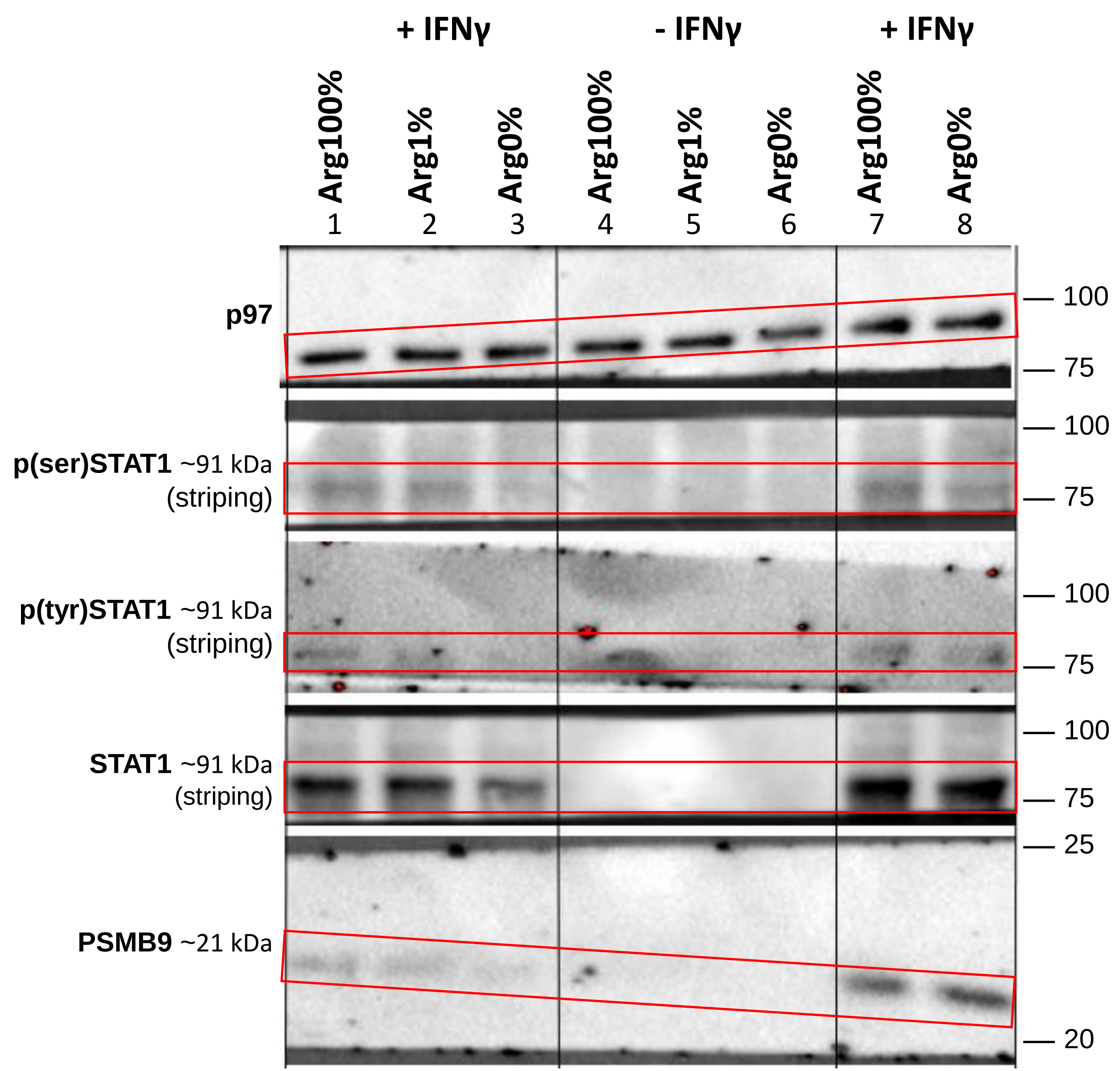

Adj. Volume (Int)

Lane	p97	p(ser)STAT1	p(tyr)STAT1	psmb9	psmb9
1	2640300	2081079	1684728	1921250	2064825
2	2860596	727461	2076960	1568544	1964160
3	2713800	217980	918384	1347736	1419525
4	2609450	159016	3386556	2101440	614664
5	2491996	35575	673623	2071250	587312
6	2196662	424400	265903	1426275	377564
7	2829680	572373	4330647	2231230	3606746
8	2787950	216864	1411410	1966250	3789760

Ratio (gene/p97)

Lane	p(ser)STAT1	p(tyr)STAT1	psmb9	psmb9
1	0.78819793	0.63808204	0.7276635	0.78204181
2	0.254304	0.72605849	0.5483277	0.68662614
3	0.08032279	0.33841256	0.4966232	0.5230765
4	0.06093851	1.29780452	0.8053191	0.23555309
5	0.01427571	0.27031464	0.831161	0.23567935
6	0.19320223	0.12104866	0.649292	0.17188079
7	0.20227482	1.53043701	0.7885097	1.27461268
8	0.07778619	0.5062537	0.7052673	1.35933571

Figure S4D – original blots

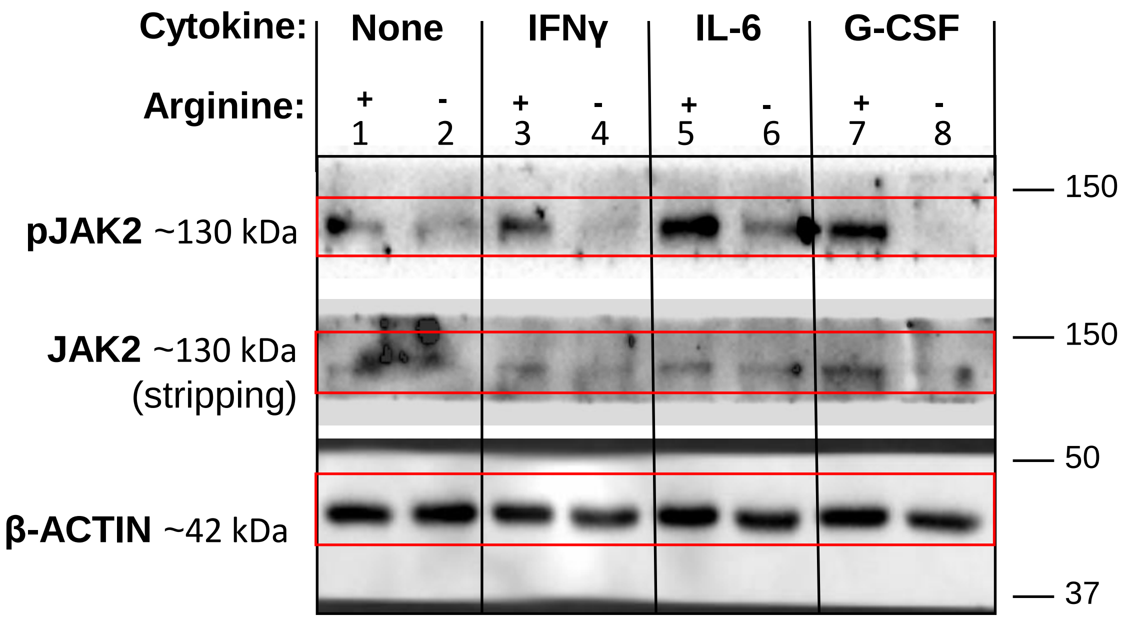

Adj. Volume (Int)

Lane	β-ACTIN	pJAK2	JAK2
1	2977889	2268087	1166123
2	3391190	1917955	260751
3	2623710	2766141	144900
4	2820686	1686699	306153
5	3312536	3862614	870345
6	3084543	3944600	645062
7	3387111	3209577	1236081
8	2838360	411730	212267

Ratio (gene/β-ACTIN)

Lane	pJAK2	Jak2
1	0.76164	0.39159
2	0.56557	0.07689
3	1.05429	0.05523
4	0.59797	0.10854
5	1.16606	0.26274
6	1.27883	0.20913
7	0.94759	0.36494
8	0.14506	0.07479
